# Phytochromes transmit photoperiod information via the evening complex in Brachypodium

**DOI:** 10.1186/s13059-023-03082-w

**Published:** 2023-11-07

**Authors:** Mingjun Gao, Yunlong Lu, Feng Geng, Cornelia Klose, Anne-Marie Staudt, He Huang, Duy Nguyen, Hui Lan, Han Lu, Todd C. Mockler, Dmitri A. Nusinow, Andreas Hiltbrunner, Eberhard Schäfer, Philip A. Wigge, Katja E. Jaeger

**Affiliations:** 1 Sainsbury Laboratory, University of Cambridge, 47 Bateman St., Cambridge, CB2 1LR UK; 2 Ministry of Education Key Laboratory for Biodiversity Science and Ecological Engineering, National Observations and Research Station for Wetland Ecosystems of the Yangtze Estuary, Institute of Biodiversity Science and Institute of Eco-Chongming, School of Life Sciences, Fudan University, Shanghai, China; 3 Leibniz-Institut für Gemüse- und Zierpflanzenbau, Theodor-Echtermeyer-Weg 1, Großbeeren, 14979 Germany; 4 Institut für Biologie II, University of Freiburg, Schaenzlestr. 1, Freiburg, 79104 Germany; 5 Donald Danforth Plant Science Center, St. Louis, MO 63132 USA; 6 Signalling Research Centres BIOSS and CIBSS, University of Freiburg, Freiburg, 79104 Germany; 7 BIOSS Centre for Biological Signalling Studies, University of Freiburg, Schaenzlestr. 18, Freiburg, 79104 Germany; 8 Institute of Biochemistry and Biology, University of Potsdam, Potsdam, 14476 Germany

## Abstract

**Background:**

Daylength is a key seasonal cue for animals and plants. In cereals, photoperiodic responses are a major adaptive trait, and alleles of clock genes such as *PHOTOPERIOD1 (PPD1)* and *EARLY FLOWERING3 (ELF3)* have been selected for in adapting barley and wheat to northern latitudes. How monocot plants sense photoperiod and integrate this information into growth and development is not well understood.

**Results:**

We find that *phytochrome C (PHYC)* is essential for flowering in *Brachypodium distachyon*. Conversely, ELF3 acts as a floral repressor and *elf3* mutants display a constitutive long day phenotype and transcriptome. We find that ELF3 and PHYC occur in a common complex. ELF3 associates with the promoters of a number of conserved regulators of flowering, including *PPD1* and *VRN1*. Consistent with observations in barley, we are able to show that *PPD1* overexpression accelerates flowering in short days and is necessary for rapid flowering in response to long days. PHYC is in the active Pfr state at the end of the day, but we observe it undergoes dark reversion over the course of the night.

**Conclusions:**

We propose that PHYC acts as a molecular timer and communicates information on night-length to the circadian clock via ELF3.

**Supplementary Information:**

The online version contains supplementary material available at 10.1186/s13059-023-03082-w.

## Background

Flowering is a major developmental transition, and plants have evolved pathways to flower in response to seasonal cues to maximize their reproductive fitness [1]. Photoperiod provides key seasonal information, and in temperate climates, long photoperiods serve as a signal of spring and summer and accelerate flowering in many plants. In *Arabidopsis thaliana*, long days (LD) result in the stabilization of the floral activator CONSTANS (CO), which activates the expression of the florigen encoding gene *FLOWERING LOCUS T (FT)* [2]. Temperate grasses, such as Brachypodium, barley and wheat also induce flowering through the induction of *FT*-related genes; however, there are differences in the signaling pathways activating *FT* expression [1, 3–6].

The major regulator of natural variation in photoperiod responsiveness in barley is the transcriptional regulator *PHOTOPERIOD1* (Hv-Ppd1), identified as a recessive allele that delays flowering under long day (LD) conditions, making plants photoperiod insensitive [6]. Natural variation of *PPD1* in wheat has led to dominant mutations in this gene that accelerate flowering [7]. Analyses of *PPD1* alleles indicate that promoter insertions and deletions have played a major role modulating *PPD1* expression, revealing a 95-bp region within the promoter that is conserved between wheat, barley, and Brachypodium [7, 8]. While this work was in review, studies in wheat have shown by chromatin immunopurification that ELF3 indeed binds and represses *PPD1* [9]. It has been hypothesized that a photoperiod-dependent repressor may bind this 95-bp region in short days to inhibit flowering. *Ppd-H1* also influences leaf size, a trait which is under photoperiod control, consistent with *Ppd-H1* being a key output of the photoperiod pathway in grasses [10].

The evening complex (EC), an integral component of the circadian clock, is also a key regulator of photoperiodism in grasses. The *early maturity8 (eam8)* allele in barley confers early flowering in SD and encodes the barley ortholog of *EARLY FLOWERING3 (ELF3)* [5], and in wheat, *Earliness Per Se (eps)* also confers early flowering and is caused by mutation in an *ELF3* related gene [11, 12]. Similarly, *eam10* encodes HvLUX, and is necessary for correctly responding to photoperiod [13], while *PHYTOCLOCK (LUX)* alleles also confer early flowering in wheat [14]. The central role of the EC in mediating photoperiod responses has recently been seen in diverse plants including Brachypodium [15, 16], rice [17], soybean [18], and maize [19].

Unlike in Arabidopsis, where phytochromes mostly repress flowering, PHYC is an essential inducer of flowering in Brachypodium [20], and interfering with PHYC in barley and wheat also greatly delays flowering, indicating that PHYC is an essential input for photoperiodism [21, 22]. Consistent with this, *phyC-1* in Brachypodium also shows additional photoperiod phenotypes such as leaf morphology differences as well as flowering time [20]. How the EC and PPD1 influence flowering and how PHYC conveys photoperiod information to these regulators is however not well understood.

## Results and discussion

**Fig. 1:**
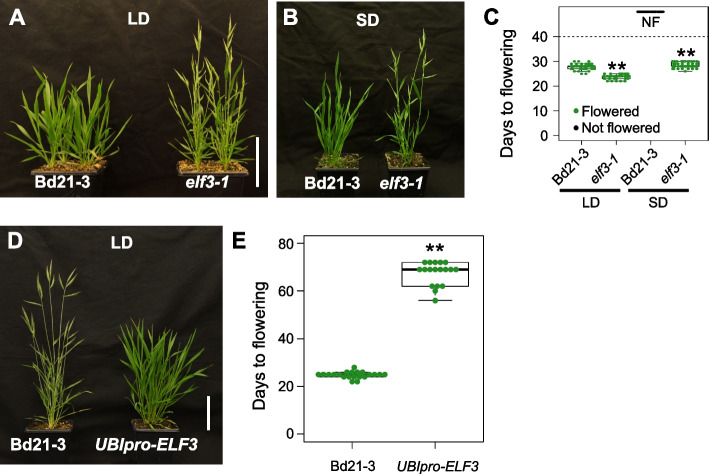
ELF3 is necessary for photoperiodism in Brachypodium. **A**–**C** elf3-1 shows a constitutive long day (20 h day:4 h night) flowering phenotype under short day conditions (12 h day:12 h night), where wild-type does not flower (NF) (Student’s *t*-test, **p-value < 0.01). **D** and **E** Constitutive expression of ELF3 under the UBIQUITIN promoter (UBIpro) is sufficient to greatly delay flowering under inductive long day conditions (Student’s *t*-test, **p-value < 0.01)

**Fig. 2:**
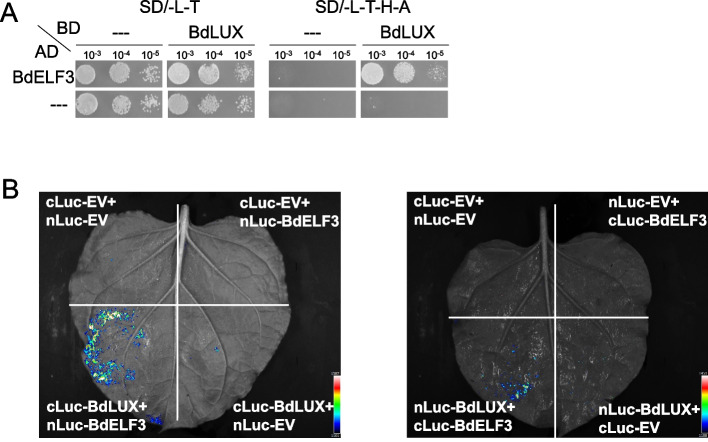
BdELF3 interacts with BdLUX. **A** BdELF3 and BdLUX interact in the yeast 2-hybrid system. Yeast transformed with BdLUX fused to the GAL4 DNA binding domain (BD) and BdELF3 with the activation domain (AD) are able to grow on –His media indicating interaction between the proteins. This interaction is dependent on the proteins, as empty vector controls (---) do not support growth on –His. **B** Split luciferase (LUC) complementation assay shows interaction between BdELF3 and BdLUX in vivo. Tobacco leaves have been infected with BdLUX fused to the C-terminal domain of LUC and BdELF3 with the N-terminal domain of LUC. This interaction is specific as empty vector controls (EV) emit no Luciferin signal

To determine if the role of *ELF3* in flowering is conserved in Brachypodium, we created loss of function alleles in *ELF3* using genome-editing. *elf3-1* plants show constitutive early flowering, largely independent of day-length, indicating that *ELF3* is necessary for responding to photoperiod (Fig. 1A–C); this is consistent with a recent study [16]. We find that *ELF3* overexpressing plants show delayed flowering in long days, suggesting that *ELF3* is necessary and sufficient to transmit photoperiodic signals in Brachypodium (Fig. 1D and E). To understand how *ELF3* may be controlling photoperiodic responses in Brachypodium, we performed affinity purification coupled with mass spectrometry to identify the ELF3 protein interactome. Consistent with the evening complex being conserved between Arabidopsis and monocots [15], ELF4 and LUX are detected as ELF3 interactors (Table 1; Additional file 1: Supplementary Dataset S1). This interaction was independently verified using the yeast-2-hybrid system (Fig. 2A) and in vivo using the split luciferase (LUC) complementation assay (Fig. 2B).

**Table 1:** ELF3 interacts with other evening complex proteins and the light signaling network in Brachypodium

Gene ID	Protein/homolog protein	Molecular weight	Exclusive unique peptide count/percent coverage^a^	
			rep1	rep2
Bradi1g64360	phyB	129 kD	32/39%	24/30%
Bradi1g08400	phyC	126 kD	2/2.8%	2/2.9%
Bradi2g00992	BED ZINC FINGER AND HAT DIMERIZATION DOMAIN-CONTAINING PROTEIN	82 kD	13/26%	11/25%
Bradi2g09080	TOPLESS	125 kD	18/22%	7/6.6%
Bradi3g16250	TOPLESS	125 kD	18/20%	10/11%
Bradi3g57667	SPA1	77 kD	13/27%	8/18%
Bradi2g46850	PCH1^b^	83 kD	10/22%	5/14%
Bradi4g07110	MYB transcription factor	110 kD	15/25%	4/5%
Bradi3g03317	Splicing factor	136 kD	16/17%	3/3.5%
Bradi1g24100	TIME FOR COFFEE	171 kD	8/10%	9/9.3%
Bradi2g62067	LUX	27 kD	3/31%	2/20%
Bradi2g48657	SPA3	87 kD	12/22%	7/14%
Bradi4g13227	ELF4	13 kD	3/33%	4/34%
Bradi1g05950	MLK1/3/4 or PPK2/3/1	79 kD	10/23%	4/9.6%
Bradi1g05950	FVE	50 kD	9/41%	4/16%
Bradi3g19927	KNOX domain-containing protein	33 kD	4/11%	4/11%

Curated list of proteins co-purified with BdELF3-GFP-FLAG which were specifically identified from affinity purification coupled with mass spectrometry (AP-MS) analyses using 45-day-old plants (old, 2 reps) or 21- day-old plants (young, 1 rep) harvested at ZT0 in dark (14 h light at 24 °C:10 h dark at 18 °C). Negative control YFP-HFC does not detect any peptides of those proteins

^a^All listed proteins match 99% protein threshold, minimum number peptides of 2, peptide threshold as 95%, and not detected in the YFP-HFC control

^b^Brachy PCH1 may has F-box domain as peptide detected in the C-terminus

The identification of two TOPLESS (TPL)-related proteins in the protein interactome suggests a mechanism by which the evening complex represses gene expression. Photoperiodism in Arabidopsis is also mediated by the repression of *FT* and *CO* by a TPL containing transcriptional complex, indicating this may be a common mechanism to achieve photoperiodic gene expression [23]. Photoperiodism requires light perception, and we identified the light sensing phytochromes PHYB and PHYC as ELF3 interactors. Brachypodium contains three phytochromes, and we therefore investigated the extent to which phytochromes are necessary for photoperiodism. *phyC-4* does not flower under LD, consistent with previous reports (Fig. S1A) [20], while *phyA-1* show delayed responses to LD (Fig. S1B). These results suggest that phytochromes act in the same pathway as *ELF3*.

**Fig. 3:**
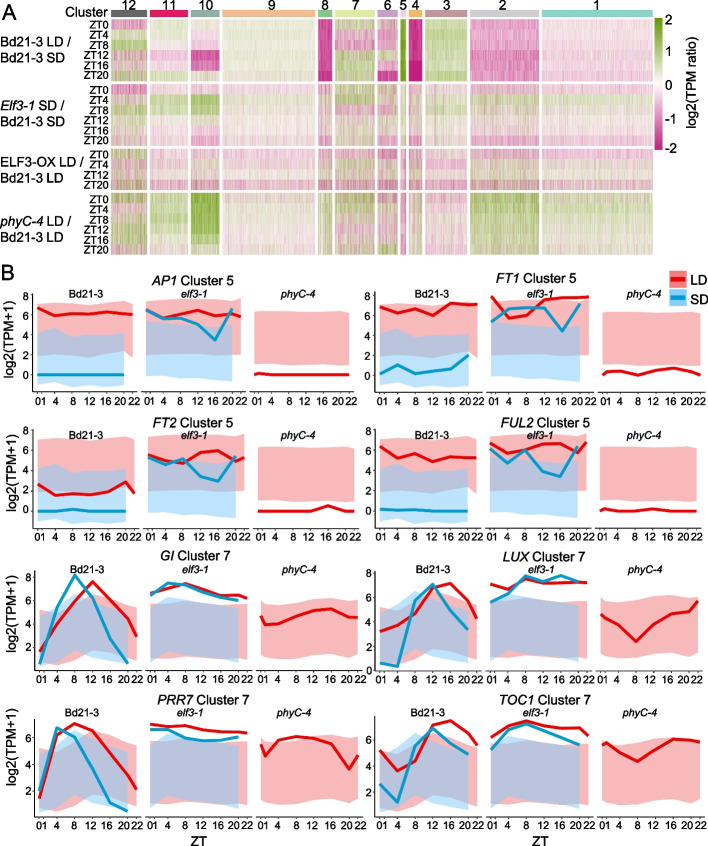
elf3-1 displays a constitutive LD transcriptome and phyC-4 resembles a SD grown plant. **A** Transcripts were clustered according photoperiod response. Multiple clusters show strong upregulation in response to LD (i.e., clusters 3, 5, and 7), while others are strongly downregulated. Man of the clusters that respond to LD are also upregulated in elf3-1 in SD. **B** Individual genes show strong photoperiod responsiveness which is dependent on ELF3. For example, clock genes such as GI, LUX, TOC1, and PRR7 are repressed at dusk in an ELF3 dependent fashion and more strongly repressed in SD than LD

To understand the broader influence of ELF3 and phyC signaling on the photoperiod response, we analyzed gene expression over 24 h in both LD and SD growth conditions (Fig. 3A; Additional file 2: Supplementary Dataset S2) [24]. Clustering of wild-type gene expression reveals prominent clusters that are repressed in response to LD (clusters 1, 2, 4, 6, 8, and 10), while other groups of genes are induced (clusters 3, 5, 7, and 9). In *elf3-1* in SD, we observe a phenocopying of the LD gene expression response, for example, clusters 3 and 5 that are up-regulated by LD are also up-regulated in *elf3*-*1* in SD. Consistent with the non-flowering phenotype of *phyC-4*, this background shows constitutive activation of SD responsive clusters even in LD. Finally, overexpression of *ELF3* in LD causes repression of the LD activated expression clusters. The importance of *ELF3* and *PHYC* for the expression of photoperiodism can clearly be seen when individual genes from representative clusters are observed directly. Consistent with the early flowering response of LD and *elf3-1* plants, we see that *FT1* and *FT2*, two florigen encoding genes, are strongly upregulated in these conditions, as is the key floral regulator *AP1 and VRN1*. The circadian regulators *GI*, *LUX*, *PRR7*, and *TOC1* show oscillating behavior in WT with repression at the end of the day, with the evening repression being stronger in SD (Fig. 3B). By comparison, oscillations and photoperiod responsiveness are abolished in *elf3-1*, with *phyC-4* showing reduced expression. These results show that *ELF3* is essential for photoperiod responses in Brachypodium.

**Fig. 4:**
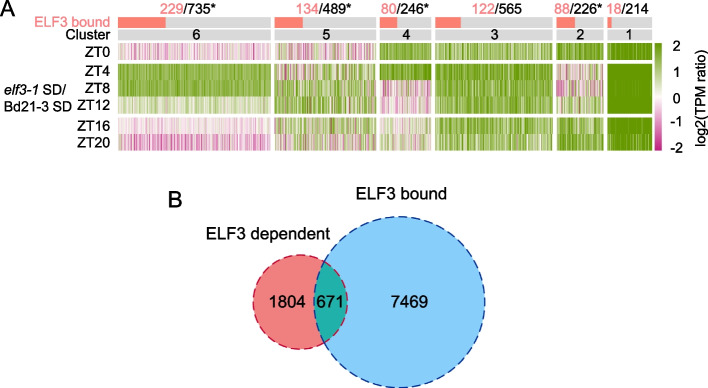
ELF3 directly controls many photoperiod responsive genes. **A** Clustering to visualize 2475 genes that are upregulated in *elf3-1* in at least two timepoints. The proportion of genes that are bound by ELF3 is shown by the orange bars (*p* < 0.05 by Fisher’s exact test denoted with *) (upregulation is defined as log2(TPM_elf3-1_SD + 1) -log2(TPM_Bd21- 3_SD + 1) > 1 in at least two timepoints). **B** 671 genes in total are both bound by ELF3 and upregulated in *elf3-1*; we define this set as the ELF3 functional targets

To understand how *ELF3* influences photoperiodic gene expression and flowering, we identified those genes that are upregulated in at least two time points in the *elf3-1* transcriptome compared to wild-type. We identified 2475 genes in this way, which fall into major clusters, depending on when they are most highly induced in *elf3-1* (Fig. 4A; Additional file 3: Supplementary Dataset S3). To identify which of these candidates are directly regulated by ELF3, we performed ChIP-seq using anti-FLAG antibody. We detect 8140 significantly bound ELF3 peaks at ZT20, with 671 genes that are both ELF3-bound and upregulated in *elf3-1* (Fig. 4B; Additional file 4: Supplementary Dataset S4) [25], and all of the *elf3-1* responsive expression clusters that show time of day specific responses are significantly enriched for ELF3 binding (Fig. 4A). Of these genes controlled by ELF3, we observe many of the genes that have been described previously as evening complex (EC) targets (Supplementary Figs. S3-S6). This includes the key circadian regulators *GI*, *LUX*, *FKF1*, *ELF4-L4*, *LNK1*, and *LNK2*, four members of the *PRR* gene family and 7 members of the B-box (BBX) class of zinc-finger transcription factors. These genes all share a common transcriptional pattern, being more highly expressed in LD and being repressed at dusk, particularly under short day conditions (Supplementary Figs. S3-S6). These photoperiod responsive ELF3 targets lose most photoperiod responsiveness in *elf3-1*, indicating that ELF3 is essential to confer photoperiodism on their expression. In addition to *LUX*, which is a common EC target in many plants, *ELF4-L4* is also directly regulated by ELF3, suggesting an additional mechanism by which the EC may control the expression of its own components (Fig. S3).

Since ELF3 has such a strong influence on flowering, we looked for target genes that may control this. *PIF4* (Bradi1g13980) is repressed by ELF3, and it loses photoperiodism becoming constitutively expressed in *elf3-1*. Since *PIF4* overexpressors in Arabidopsis are very early flowering [26, 27], and PIF4 plays a role in integrating environmental signals to coordinate flowering, this represents an interesting candidate for accelerating flowering in Brachypodium. Another positive regulator of flowering in Arabidopsis is the clock gene *GIGANTEA (GI) *[28], and we see this is also directly repressed by ELF3 and becomes upregulated under inductive photoperiods in Brachypodium. The BBX genes are a major class of transcriptional regulators, many of which are involved in flowering control in monocots and Arabidopsis. For example, the key regulator of photoperiodism in Arabidopsis is *CONSTANS (CO/BBX1)*. Since 7 BBX genes are responsive to photoperiod and directly regulated by ELF3, this family may also play a central role in flowering responses in Brachypodium.

The floral transition in wheat, barley, and Brachypodium is controlled by the key MADS box transcription factor VRN1 [29–33]. *BdVRN1* knockdown lines are late flowering, and higher levels of *BdVRN1* expression are associated with early flowering [30, 33, 34]. We observe direct control of *VRN1* by ELF3, as well as the *AP1-*related genes Bradi1g21980 and Bradi1g08340 (Fig. S6), Bradi1g77020 (related to *SOC1*), and Bradi2g59191 (*AGL19*). We observe direct regulation of the two circadian regulators *NIGHT LIGHT-INDUCIBLE AND CLOCK-REGULATED GENE 1 (LNK1)* and *LNK2*. In Arabidopsis, *lnk1 lnk2* double mutants are late flowering in long days [35], suggesting this role may be conserved in Brachypodium.

**Fig. 5:**
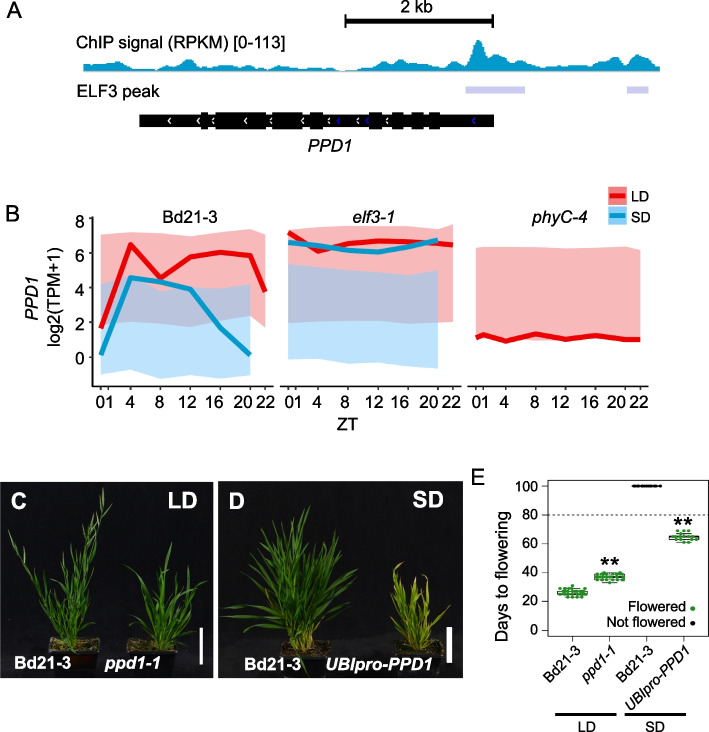
ELF3 directly controls the flowering regulator *PPD1*. **A** ELF3 associates with the promoter of *PPD1* as measured by ChIP-seq. **B** *PPD1* expression is strongly repressed during the night in SD in an *ELF3*- dependent manner. *PPD1* remains stably suppressed in the *phyC* background. **C**–**E** *ppd1-1* is late flowering in LD conditions, while overexpression of *PPD1* under the ubiquitin (*UBI*) promoter can rescue non-flowering in SD

A key regulator of flowering time in monocots is the *PRR37/PPD1* class of genes. We observe that *PPD1* (Bradi1g16490) is strongly repressed by ELF3 in the evening in a photoperiod specific manner (Fig. 5A and B). To determine if *PPD1* serves as a regulator of photoperiodism in Brachypodium, we created a loss of function allele, *ppd1-1*. This line shows delayed flowering in long days, indicating it is necessary for acceleration of flowering in response to inductive conditions (Fig. 5C and E). ELF3 binds directly to *PPD1* (Fig. S4), and it is likely that the regulation of *PPD1* expression by ELF3 is important, since overexpressing *PPD1* under the *UBIQUITIN* promoter is sufficient to trigger flowering in non-inductive short days (Fig. 5D and E). The *ppd1-1* transcriptome shows a similar behavior to that of *phyC-4*, and many of the targets of the ELF3 repressed target genes are repressed in *ppd1-1* (Additional file 10: Fig. S7 and Additional file 8: Supplementary Dataset S8). Since *ppd1-1* does not have as strong a flowering phenotype as *phyC-4*, this indicates that other directly regulated ELF3 targets such as the other *PRR* genes, *VRN1* and the *AP1/SOC1* homologs, the *BBX* genes, *GI*, *LNK1*, and* 2*, and perhaps *PIF4* also contribute to the ELF3-photoperiod flowering response. A recent study has shown that an independent allele of *PPD1* has a more delayed flowering phenotype [36], suggesting that the *ppd1-1* allele generated in this study may be a weaker hypomorph.

**Fig. 6:**
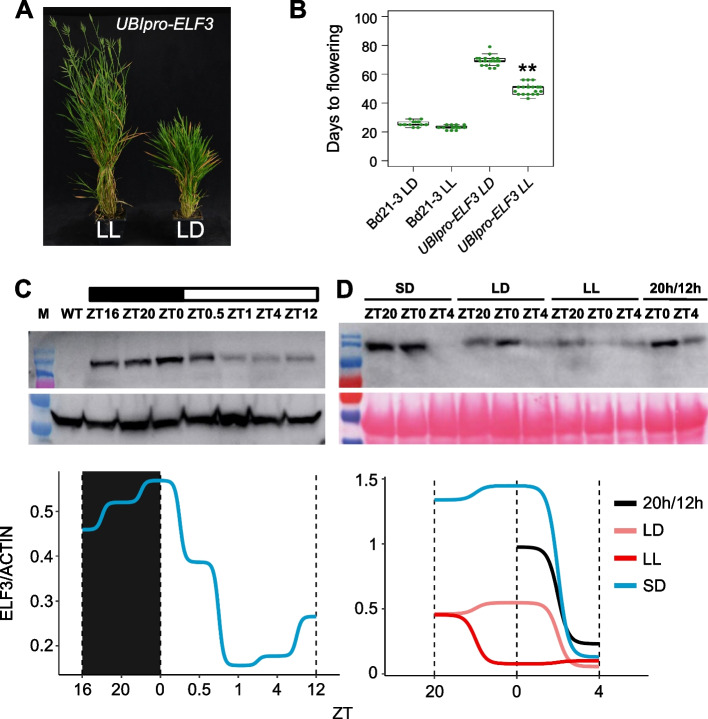
ELF3 protein levels integrate photoperiod information. **A**–**B** The late flowering phenotype of *UBIpro-ELF3* in long days is partially suppressed by growth in continuous light. **C**, **D** ELF3 protein levels accumulate during the night and are rapidly reduced on exposure to light (plants were grown under 12 h dark:12 h light regime), blot was probed with anti M2 FLAG antibody (**C**, **D**) or anti ACTIN antibody for normalization of band intensity (**C**)

These results show that ELF3 is a major central integrator of the flowering response since it binds to the promoters and regulates the expression of many flowering regulators such as *PPD1*, *GI*, and *VRN1* (Figs. 4 and 5, S3 to S6). Under inductive LD photoperiods, ELF3 levels decline, enabling the upregulation of these transcriptional activators and the initiation of flowering (Fig. 6D). The expression of *ELF3* however is largely constant and does not show significant circadian variation, and it is expressed in both SD and LD (Fig. S8). This suggests that the regulation of *ELF3* may be post-translational. Consistent with this hypothesis, the late flowering phenotype of *UBI-ELF3* is sensitive to light exposure (Fig. 6). While *UBI-ELF3* plants never flower in SD, they are very late in LD, but flowering is accelerated under continuous light exposure (LL), suggesting that light exposure influences ELF3 activity. Indeed, ELF3 protein accumulates at the end of the night to high levels under SD, and is rapidly degraded upon exposure to light, which is consistent with recent reports in wheat [37]. A similar pattern is seen under LD, but the levels of ELF3 are lower (Fig. 6C and D). ELF3 protein is not detectable in Bd21-3 wild-type background but is able to accumulate in the *phyC-4* background during the day (ZT4) (Fig. S9).

**Fig. 7:**
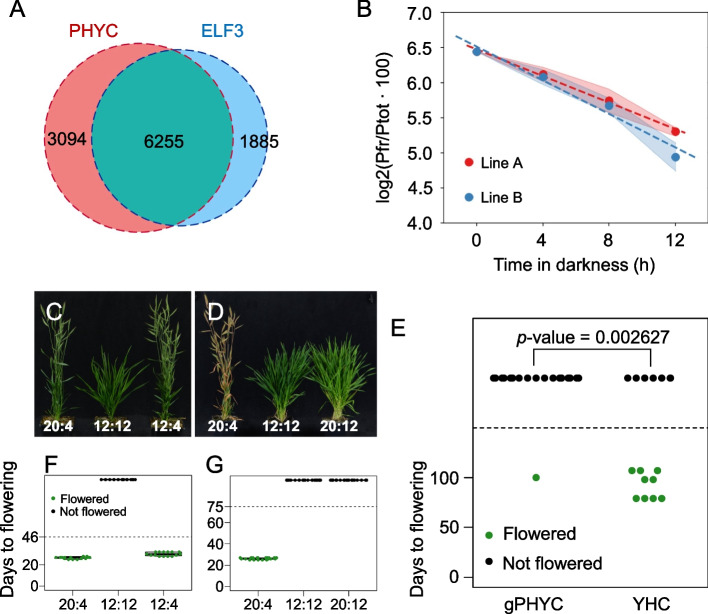
ELF3 protein levels integrate photoperiod information. **A** Overlap between PHYC and ELF3 bound genes. **B** PHYC dark reversion has a half-life of about 8 h. Line A, *y* =  − 0.094x + 6.476 (linear regression function), BdPHYC in Arabidopsis *phyAB* double mutant background; Line B, *y* =  − 0.12x + 6.515 (linear regression function), BdPHYC in *B. distachyon* WT background. **C**–**D** Night length but not day length is the key determinant of when plants will flower. Numbers represent day length (hour): night length (hour). **E** The *YHC* mutation restores flowering under short day conditions. *p*-value was calculated by chi-squared test; the experiment was terminated after 100 days. YHC, Y242H point mutation in PHYC. gPHYC, genomic PHYC

Since *phyC-4* transcriptionally resembles a plant with elevated ELF3 signaling, this suggests that PHYC may be the major light receptor controlling ELF3 activity. To determine if this occurs via a direct mechanism, we performed ChIP-seq of PHYC. In Arabidopsis, PHYB binds to target genes to modulate their expression [38], and we investigated if this might be true for PHYC. We observe coincidence between ELF3 and PHYC ChIP-seq peaks for many key genes such as *LUX* (Fig. 7A; Additional file 6: Supplementary Dataset S6). Phytochromes have been observed to interact with ELF3 in other systems [9, 39, 40]. These results are in agreement with a recent study showing that in Brachypodium *phyC elf3* double mutants showed a restored early flowering phenotype compared to the *phyC* single mutant [36]. Our results suggest PHYC suppresses the ability of ELF3 to repress its target genes.

Since phytochromes in the active, Pfr, state slowly revert to the inactive Pr state in the dark (thermal or dark reversion), we hypothesized that this presents a mechanism for measuring the length of the night. Under long photoperiods, the dark period may be insufficient for PHYC Pfr to be depleted, with the result that ELF3 cannot accumulate to a high level. Extending the night period in short days however may enable PHYC Pfr to become depleted, allowing the accumulation of repressive ELF3. To test this, we measured the dark reversion dynamics of Brachypodium PHYC by overexpressing the gene in Brachypodium and Arabidopsis seedlings. In both cases, we observe similar reversion rates, and the dark reversion of PHYC Pfr has a half-life of 8.3 h in Brachypodium (Fig. 7B; Supplementary Fig. S10). This indicates that the Pfr dark reversion rate is suitable to distinguish between long and short photoperiods by measuring the length of darkness.

These results suggest that unlike in the case of Arabidopsis in which daylength is measured to contribute photoperiodic flowering [2], Brachypodium may measure the length of the night to determine photoperiodism. To test this directly, we used non 24 h day night cycles to determine whether the length of the night or day is more important for flowering. While a SD (12 h:12 h, day to night) is non-inductive, flowering is accelerated simply by reducing the length of the night in 12 h:4 h photoperiods (Fig. 7C, F, S2A). By contrast, a 20-h day is unable to trigger flowering when coupled with a long night (Fig. 7D, G, S2A). These results suggest that the rate of dark reversion is an important component in responding to night-length. To confirm this, we engineered a version of PHYC which contains the point mutation that has been shown to prevent dark-reversion in phyB in Arabidopsis [41]. This stabilized version of PHYC (YHC) is predicted to not undergo dark reversion, enabling it to maintain activity in darkness and trigger flowering even in SD. In agreement with this hypothesis, the majority of plants expressing YHC flower within 100 days under non-inductive SD conditions, while only a single wild-type control plant expressing *PHYC* did (Fig. 7E).

Brachypodium therefore appears to use night length to infer photoperiod. This is likely a common mechanism for monocots, since night-break experiments perturb flowering in Brachypodium, wheat and rice [16, 42, 43], and PHYC is an important regulator of photoperiodic responses in cereals [20, 21, 44]. To test this directly, we conducted a night break experiment (NB) and show that NB promotes flowering in a 12L:12D photoperiod (Fig. S2B). Rice is a short-day plant, but it appears that the genetic interactions between OsELF3-1 and phytochromes in controlling photoperiodism are conserved [45].

## Discussion

**Fig. 8:**
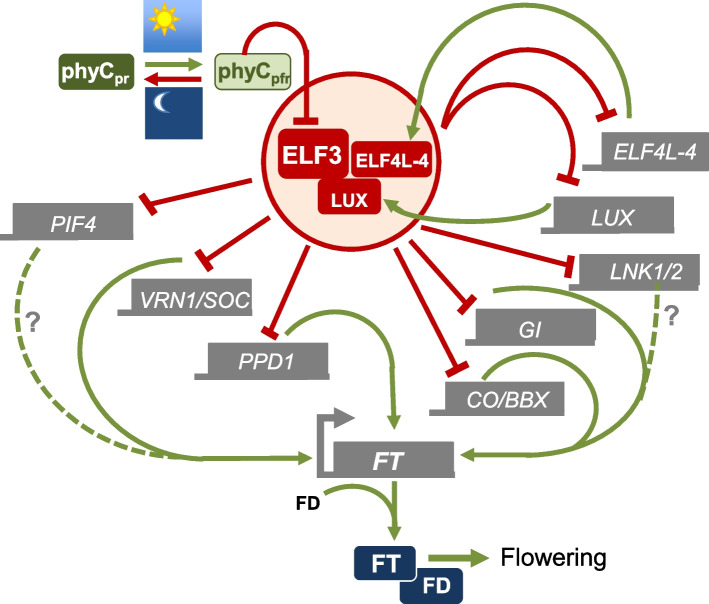
The EC integrates photoperiod information to control flowering in Brachypodium. Activity of ELF3 is controlled by light via phytochromes, particularly phyC. Long photoperiods lead to long periods of phyC activation and the inactivation of ELF3 and the EC. The EC auto-regulates its own activity by repressing LUX and ELF4L-4. The EC controls flowering by directly repressing the expression of key positive flowering regulators, including VRN1, 3 related MADS transcription factors, PPD1, GI, LNK1, and 2 and several members of the BBX family. Under long photoperiods, the accumulation of active phyCpfr results in the reduction of EC activity and upregulation of many floral activators, leading to the increase of FT expression and flowering. Genes are shown with grey boxes, and protein products in rectangles with rounded corners

It has recently been shown that the EC controls photoperiodism in rice, and this is mediated through phytochrome signaling [17]. Since photoperiodism is also controlled by an *ELF3* ortholog in Pea, this pathway appears to be broadly conserved [46]. While it had been proposed that the phytochrome dark reversion rate could serve as a molecular “hourglass,” providing a timer function for photoperiodism [47], this model was discounted on discovering a circadian variation in sensitivity to far red-light pulses during extended darkness [48]. Our finding that phytochromes directly modulate the activity of the circadian component ELF3 suggests a mechanism to reconcile these observations (Fig. 8). Light quality at dusk varies seasonally [49, 50], and in Aspen phytochrome signaling controls growth cessation and bud set during autumn [51]. The ability of phytochromes to integrate changes in both spectral quality and photoperiod may represent a robust mechanism for making seasonal decisions.

## Conclusions

Photoperiod information is transmitted via phytochrome signaling to directly control the activity of ELF3 in *Brachypodium distachyon*. ELF3 serves as a major integrator of circadian and environmental signaling and directly regulates the expression of many key flowering genes, including *LNK1*, *LNK2*, *GI*, *CO*, *PPD1*, and *VRN1/SOC1*.

## Methods

### Plant materials and growth conditions

*Brachypodium distachyon* accession *Bd21-3* was used in this study. Seeds were imbibed in distilled water at 4 °C for 2 days before sowing. Plants were grown in 5 parts John Innes #2, 3 parts peat, 1 parts silver sand, 3 parts course vermiculite, Osmocote 2.7 g/L. All plants were grown in growth cabinets with constant temperature 20 °C, 65% humidity, and 350 μmol m^−2^ s^−1^ PPFD (Photosynthetic Photon Flux Density). For flowering-time experiments, plants were grown in photoperiod regimes: (a) LD (20 h day/4 h night); (b) SD (12 h day/12 h night); (c) 20:12 (20 h day/12 h night); (d) 12:4 (12 h day/4 h night).

### Mutants used in this study

**Table:** 

**Line name**	**Background**	**Description**	**Source**	**Notes**
*elf3-1*	Bd21-3	crispr line	This study	7 bp deletion or 1 bp insertion in the second exon, both caused premature stop codon
*phyC-4*	Bd21-3	crispr line	This study	4 bp deletion in the first exon, caused premature stop codon
*ppd1-1*	Bd21-3	crispr line	This study	1 bp deletion in the sixth exon, caused premature stop codon
*phyA-1*	Bd21-3	crispr line	This study	3 bp deletion in the first exon, caused one amino acid deletion
*UBIpro-ELF3-GFP-Flag*	Bd21-3	transgenic line	This study	
*UBIpro-PPD1-GFP_Flag*	Bd21-3	transgenic line	This study	

The *phyC-1 EMS* mutant has been described previously [20]. For this study, we created CRISPR mutation in the *ELF3* gene (Bradi2g14290), *PHYC* gene (Bradi1g08400), and *PPD1* gene (Bradi1g16490). The cloning of the single-guide RNA (sgRNA) was done as described in [52]. sgRNAs primers for *ELF3*, *PPD1*, and *PHYC* were designed using design tool http://www.e-crisp.org/E-CRISP/. The annealed oligos were ligated into entry vector pOs-sgRNA and then cloned into destination vector pH-Ubi-cas9-7 by gateway LR reaction. The constructs were transformed in the *Agrobacterium* strain AGL1. *Agrobacterium*-mediated plant transformation of embryonic callus generated from immature embryos was performed as described [53]. For the genotyping analysis, mutations were confirmed by sequencing and T2 lines with mutation but not carrying Hyg resistance and were selected for further analysis.

For the overexpressing transgenic lines, the genomic coding sequence of *ELF3*, *PPD1*, and *PHYC* were amplified by PCR with primers indicated in Table S1. The PCR products were cloned into SLIC binary vector containing ubiquitin promoter and C-terminal 3xFLAG tag using NEBuilder® HiFi DNA Assembly Cloning Kit (E2621L). pENTR-YFP-His_6_-3xFLAG [54] was recombined using the Gateway system (Invitrogen) into pMDC32 [55]. Embryogenic calli from *B. distachyon* 21-3 plants were transformed with pMDC32-YFP-His_6_-3xFLAG as described [56]. For each construct, approximately 20 independent transgenic lines were obtained and homozygous single insertion lines were selected for further analysis.

For overexpression of *PHYC* in Arabidopsis, the *PHYC* genomic fragment was amplified and then cloned into 35S and N-terminal 3xFLAG tagged binary vector by NEBuilder® HiFi DNA Assembly Cloning Kit (E2621L). The binary construct was transformed into Arabidopsis *phyAB* mutant by floral dipping method. The *35S-N3FLAG-PHYC* transgenic plants were isolated by Kanamycin selection and propagated to obtain homozygous seeds to measure the dark reversion rate. Altering of the GAF (Tyr-to-His) of phyB of *Arabidopsis thaliana* resulted in photoinsensitive mutant alleles of phytochrome B (*PHYBY276H*, *or YHB*) [57]. Therefore, we aligned phyC from Brachypodium with YHB of *Arabidopsis* and identified the conserved GAF domain. YHC was created by overlapping PCR with phyC genomic construct as template, changing amino acid at position 242 from tyrosine to histidine, and using NEBuilder HiFi DNA Assembly Master Mix (NEB, E2621L); this PCR fragment was subsequently cloned into the pUBI vector and sequenced. This construct was transformed into callus and selected with hygromycin and plants later confirmed with PCR and by Western blot. Primers used in this study are listed in Additional file 9: Supplementary Dataset S9.

For *Western blot assay*, seeds were sterilized and sown on ½ X Murashige and Skoog-agar (MS-agar) plates at pH 5.7 and grown in Magenta™ GA-7 Plant Culture Box (Thomas scientific). Sterilized seeds were stratified for 2 days at 4 °C in the dark and allowed to germinate. The plates were transferred to short-day conditions (12 h light and 12 h dark) and collected at the indicated time.

One hundred milligrams of frozen plant material was grinded and then added 100 μl 2 × Laemmli buffer (S3401, SIGMA). The protein was denatured at 96 °C for 10 min. Fifteen microliters of protein samples were separated on 12% SDS-PAGE and blotted 7 min to nitrocellulose membrane using Turbo semi-dry transfer. Blots were blocked with 5% milk for 1 h at RT (room temperature) and then incubated in the anti-FLAG M2 (Sigma) primary antibody at a dilution of 1:2500 at 4 °C overnight with agitation or custom anti-ELF3 antibody (Agrisera, AS184168, lot# 1808) at a dilution of 1:1000 at 4 °C overnight with agitation. The antibody solution was decanted, and the blot was rinsed briefly twice, then washed once for 15 min and 3 times for 5 min in TBS-T at RT with agitation. Blot was incubated in secondary antibody goat anti-mouse IgG-HRP conjugate (Bio-Rad, #1721011) diluted to 1:5000 in for 2 h at RT with agitation or Agrisera Antibody, AS184168, lot# 1808). The blot was washed as above and developed by PiecreTM ECL substrate (Thermo Scientific, #32134). Exposure time was 15 and 30 s.

### Yeast two-hybrid (Y2H)

For the Y2H assay, the coding sequences of *BdELF3* and *BdLUX* were amplified with gene specific primers (Additional file 9: Supplementary Dataset S9) and cloned into the yeast expression vectors pGADT7 and pGBKT7. The resulting constructs were co-transformed into yeast strain AH109. The yeast transformants were grown on nutrient-restricted mediums to assess interactions between various protein combinations.

### Split luciferase complementation

The coding sequences of *BdELF3* and *BdLUX* were was amplified and cloned into pCAMBIA-35S-nLuc and pCAMBIA-35S-cLuc, respectively. The resulting plasmids were transformed into *Agrobacterium* GV3101. After culture overnight at 28 °C, the bacteria were collected and resuspended in infiltration buffer (10 mM MgCl_2_, 10 mM MES, 150 mM acetosyringone, pH 5.6) and incubated for 2–3 h at 30 °C. The suspensions were infiltrated into leaves of 3-week-old *N. benthamiana.* Luciferase activity was measured with Luciferase Assay Systems (Promega) after 2 days of transformation.

### Creating YHC transgenic plants

According to the function of YHB in Arabidopsis [57], we created the Y242H point mutation in PHYC (YHC) which is supposed to prevents the dark reversion reaction, locking PHYC in the active Pfr state. The mutation sites were introduced using two overlapping primers as listed in Additional file 9: Supplementary Dataset S9. The PCR products were cloned into SLIC binary vector. The resulting YHC constructs were transformed into Bd21-3 plants to produce more than 10 independent lines for further analysis.

### Gene expression by RNA-seq

RNA-seq experiments were performed for Bd21-3, *elf3-*1, *UBIpro:ELF3*, *phyC-4*, *ppd1-1*, and UBIpro:PPD1 at LD and SD over a 24 h timecourse. Two- or 3-week-old seedlings of the indicated genotypes were grown at 20 °C and sampled at intervals over the diurnal cycle: ZT = 0, 4, 8, 12, 16, 20, and 22 h.

Qiagen RNeasy Mini Kit (74104) was used to extract RNA. RNA quality and integrity were assessed on the Agilent 2200 TapeStation system. Library preparation was performed with 1 μg total RNA using the NEBNext® Ultra™ Directional RNA Library Prep Kit for Illumina® (E7420L). The libraries were sequenced on a NextSeq500 (Illumina) running a final pooled library. Each pool contained 24 to 30 samples and was sequenced using NextSeq® 500/550 High Output Kit v2 (150 cycles) TG-160-2002 on a NextSeq500 (Illumina).

*Q-PCR* was performed on a Roche Lightcycler using standard *reverse transcriptase* kit and SYBR Green Real-Time PCR Master Mixes (SIGMA).

### RNA-Seq data processing

Adapters were trimmed off from raw reads with Trimmomatic (v0.32) [58] with argument “ILLUMINACLIP:$FA_ADAPTER:6:30:10 LEADING:3 TRAILING:3 MINLEN:36 SLIDINGWINDOW:4:15.” Clean reads were mapped using *hisat2* (v2.0.5) [59] with argument “--no-mixed --rna-strandness RF --dta --fr.” Duplicate reads were removed with *Picard* (v1.103) [60] using default setting. Transcripts were quantified with *StringTie* (v1.3.3b) [61] in TPM values (Transcripts per Million mapped transcripts) with argument “--rf” directed by annotation version “Bdistachyon_314_v3.1” (https://phytozome-next.jgi.doe.gov/info/Bdistachyon_v3_1).

### RNA-Seq clustering

Mean TPM values were transformed into log2(TPM + 1). Genes with the maximum log2(TPM + 1) > 2 were kept. To investigate transcriptomic response towards a particular treatment, timecourse perturbation matrices were constructed as the difference of log abundances between paired conditions. For example, $log2\left(\frac{{TPM}+1}{{TPM}+1}\right)$, the selected perturbation matrices will be as follows:

- [LD/SD, WT, ZT00]
- [LD/SD, WT, ZT04]
- [LD/SD, WT, ZT08]
- [LD/SD, WT, ZT12]
- [LD/SD, WT, ZT16]
- [LD/SD, WT, ZT20]

Gaussian Mixture Models (GMM), a distribution-based clustering method and implemented by an R package *clusterR()* (https://github.com/mlampros/ClusterR), was used for performing the clustering. The expectation-maximization algorithm was used for fitting GMM to the given matrices. The Bayesian information criterion was used for estimating the number of clusters.

### ChIP-seq experimental details

Seeds were sterilized and sown on ½ X Murashige and Skoog-agar (MS-agar) plates at pH 5.7 and grown in Magenta™ GA-7 Plant Culture Box (Thomas scientific) and harvested at the indicated time.

Three-gram seedlings for each set were fixed under vacuum for 20 min in 1xPBS (10 mM PO_4_^3−^, 137 mM NaCl, and 2.7 mM KCl) containing 1% formaldehyde (F8775 SIGMA). The reaction was quenched by adding glycine to a final concentration of 62 mM. Chromatin immunoprecipitation (ChIP) was performed as described [62], with the exception that 100 μl FLAG M2 agarose affinity gel antibody was used (SIGMA-Aldrich) per sample. Sequencing libraries were prepared using TruSeq ChIP Sample Preparation Kit (Illumina IP-202-1024) and samples sequenced on NextSeq500 machine from Illumina using NextSeq® 500/550 High Output Kit v2 (75 cycles) TG-160-2005. Sequence reads were analyzed using in-house pipelines.

### ChIP-Seq data processing

For processing ChIP-seq fastq files, *bwa* (v0.7.17-r1188) was used to map raw reads to Brachypodium genome Bdistachyon_314_v3.1. Unmapped reads, mate unmapped reads, non-primary alignment, and duplicate reads were removed. Peaks were identified using *MACS2* (v2.2.7.1) and filtered by *q*-value < 0.01. Bigwig files for IGV tracks were generated using deeptools function *bamCoverage* and normalized using RPKM.

### Defining ELF3 and phyC bound genes

ELF3 bound genes were determined if ELF3 peaks overlap with the genomic regions of gene body extended by 2 kb towards upstream and downstream.

### Finding Arabidopsis homologs for Brachypodium genes

Gene names used in this study can be found in Additional file 7: Supplementary Dataset S7. BLAT on proteins was used to find Arabidopsis homologs for Brachypodium genes with thresholds identity > 40% and *E*-value < 0.05 (Additional file 8: Supplementary Dataset S8).

### Availability

Code is available from https://github.com/shouldsee/pipeline-rnaseq-hisat2-stringtie and https://github.com/yl-lu/Brachypodium_EC.

RNA-Seq and ChIP-seq data are available from Gene Expression Omnibus (GEO): GSE147373 [63], GSE128206 [64].

### Assaying dark reversion rate for PHYC

#### Lines used

- Line A: pUBI-BdPHYC-OX in an Arabidopsis *phyAB* mutant background (plant 5).
- Line B: pUBI-BdPHYC-OX 19-7 (homozygous) in *B. distachyon* WT background.

#### Method

*B. distachyon* seeds were incubated between 2 sheets of wet filter paper for 2–3 days in darkness at 4 °C. After removal of the lemma, the seeds were plated on ½ MS agar supplemented with 5 μM Norflurazon to inhibit greening during the red light irradiation. The seedlings were grown for 6 days at 22 °C in darkness. In order to induce the degradation of PHYA and PHYB, the seedlings were irradiated with constant red light (660 nm, 10 μmol m^−2^ s^−1^) for 16 h. Subsequently, the seedlings were transferred to darkness at 22 °C to monitor dark reversion of PHYC. At time points 0, 4, 8, and 12 h after dark transfer, relative levels of active PHYC (Pfr/Ptot) were measured using a dual wavelength ratio spectrophotometer (Ratiospect) as described previously [65]. The shoot parts of 5–7 *B. distachyon* seedlings were used per measurement. To inhibit oxidation, the seedlings were incubated for 20 min in ice-cold 50 mM DTT solution prior to the measurement.

*A. thaliana* seeds were sterilized before plating them on 4 layers of Whatman® filter paper saturated with 4.5 ml ddH_2_O. For sterilization, the seeds were washed first shortly with 70% ethanol and then twice with 100% ethanol. The seeds were stratified for at least 2 days at 4 °C in darkness. To induce germination, the seeds were incubated during 4 to 8 h in white light at 22 °C. Subsequently, the seedlings were grown in darkness at 22 °C for 4 days. Prior to Ratiospect measurements, the seedlings were irradiated for 20 min with constant red light (660 nm, 10 μmol m^−2^ s^−1^) to convert PYHC into the active Pfr form. Afterwards, the seedlings were transferred into darkness at 22 °C to monitor dark reversion of PHYC. At time points 0, 4, 8, and 12 h after red light irradiation, relative levels of active PHYC (Pfr/Ptot) were measured using a dual wavelength ratio spectrophotometer (Ratiospect) as described previously [65]. One hundred twenty to 140 mg of *A. thaliana* seedlings (freshweight) were used per measurement.

### Proteomics

Plant materials for affinity purification coupled with mass spectrometry (AP-MS) were made from Brachypodium plants expressing either pUBI-ELF3-GFP-FLAG or pMDC32-YFP-His_6_-3xFLAG (negative control) transgene. After stratification in dark at 4 °C for 4 days, transgenic Brachypodium plants were grown in a growth chamber with a photoperiod of 14 h of light (200 umol·m^−2^·s^−1^) and 10 h of darkness, at 24 °C during daytime and 18 °C at night. Leaves from 45-day-old (old) or 21-day-old (young) plants were harvested at ZT0 in darkness and flash frozen in liquid N_2_. The protein extraction was performed in darkness with dim green safety light. About 30 mg (for old plants sample and YFP negative control) or 10 mg (for young plants sample) of total protein were used for purification via FLAG immune-precipitation (we used 1.4 μg anti-FLAG antibody per 1 mg total protein), using the method as previously described [54, 66]. After elution with 3xFLAG free peptides, eluates were precipitated by 25% TCA at −20 °C, pelleted, and washed with ice-cold acetone. Pellets were dried using a speed vacuum and sent for mass spectrometry analysis, with the same processing protocol and filtering criteria as described previously [40]. MS data were extracted and searched against Brachypodium database to identify each protein (Phytozome 12, https://phytozome.jgi.doe.gov/pz/portal.html). All proteins identified in YFP control were subtracted from the identifications and a curated list containing ELF3 specific interactors was presented, showing names of their Arabidopsis homolog proteins.

### Supplementary Information

**Additional file 1: Supplementary Dataset S1.** Proteins Co-Purified by BdELF3-GFP-FLAG AP-MS.

**Additional file 2: Supplementary Dataset S2.** Gene expression analyzed over 24 h in both LD and SD growth conditions.

**Additional file 3: Supplementary Dataset S3.** Genes identified, which fall into major clusters.

**Additional file 4: Supplementary Dataset S4.** Significantly bound ELF3 peaks at ZT20.

**Additional file 5: Supplementary Dataset S5.***ppd1-1* transcriptome.

**Additional file 6: Supplementary Dataset S6.** Coincidence between ELF3 and PHYC ChIP-seq peaks.

**Additional file 7: Supplementary Dataset S7.** Gene names used in this study.

**Additional file 8: Supplementary Dataset S8.** BLAT on proteins.

**Additional file 9: Supplementary Dataset S9.** Primers used in this study.

**Additional file 10: Fig. S1.** Phytochromes are necessary for LD activation of flowering. A. *phyC-4* does not flower in inductive conditions. B. *phyA-1* is late flowering in long days. **Fig. S2.** Night length determines flowering phenotype in Brachypodium and a night break promotes early flowering A. Plants grown under 12L:4D condition flower nearly at the same time as plants grown und 20L:4D (LD) conditions at after about 3 weeks, whereas plants grown under 12L:12D, 20L:12D or 12L:20D did not flower during the course of the experiment. Experiment was terminated after 75 days, as plants started to senesce. A night break triggers flowering under non inductive short day conditions in Brachypodium. B. Introducing a night break of 1h or 2 nigh breaks of 30min each leads to a flowering phenotype similar to plant being grown under inductive long day conditions (growth condition set up was: 2*1h NB: 12 hour light + 4 hours dark+1hour light+3 hours dark+ 1hour light+3 hours dark. 2*0.5h NB: 12 hour light + 4 hours dark+0.5 hour light+3.5 hours dark+ 0.5 hour light+3.5 hours dark1*1h NB: 12 hour light + 6 hours dark+1hour light+5 hours dark, all Bd21). **Fig. S3.** Transcriptional and ELF3 bound pattern of representative genes. RPKM was used to show ChIP signal in IGV screenshots. **Fig. S4.** Transcriptional and ELF3 bound pattern of representative genes. RPKM was used to show ChIP signal in IGV screenshots. **Fig. S5.** Transcriptional and ELF3 bound pattern of representative genes. RPKM was used to show ChIP signal in IGV screenshots. **Fig. S6.** Transcriptional and ELF3 bound pattern of representative genes. RPKM was used to show ChIP signal in IGV screenshots. **Fig. S7.** *ppd1-1* transcriptome shows a similar behavior to *phyC-4*. A. Transcripts were clustered according photoperiod response, same with Fig. 2A, B. Transcriptional pattern of selected genes in Bd21-3, *ppd1* and *PPD1* OX under SD and LD. **Fig. S8.** ELF3 protein is degraded in response to light. Independently of photoperiod Plants were grown under 12L:12D (SD) or 20L:12D condition as indicated and samples taken 12 DAG at the indicated time (ZT20, ZT0 and ZT4, with 3 plants used per sample). We used wild type plants (lane 2, ZT0) or plants overexpressing *ELF3* (*pUBI:ELF3_GFP_FLAG*) (lane 3 to lane 7) and probed with an antibody raised in rabbit against ELF3 peptide (Agrisera AS184168, lot# 1808). **Fig. S9.** ELF3 protein is stabilized in *phyC-4* A. Plants were grown under 20L:12D condition samples taken 12 DAG at the indicated time (ZT6, with 3 plants used per sample). We used Bd21-3 (lane 2, 3), *phyC-4* (lane 4, 5) or plants overexpressing *ELF3* (*pUBI:ELF3_GFP_FLAG*) (lane 5) and probed with an antibody raised in rabbit against ELF3 peptide (Agrisera). ELF3 accumulates in *phyC-4* background at the end of the long day, but can not be detected in Bd21-3 background. ELF3 was detected using custom anti-ELF3 (Agrisera, AS184168, lot# 1808). B. Transcript level of ELF3 in phyC background are unchanged, indicating that phyC controls ELF3 in the protein level. **Fig. S10.** A. Western blot for 2 independent lines overexpressing *pUBI:phyC-GFP-Flag* in Brachypodium wild type background. B. Western blot for 6 independent lines overexpressing *35S-NFlag-BdPHYC* in Arabidopsis background. Western blot was probed with an antibody against Flag epitope (M2, Sigma). Plants were grown under 20L:4D (LD) condition and samples taken 12 DAG at the indicated time (ZT20, ZT0 with 3 plants used per sample).

**Additional file 11.** Review history.
