# Supplementary material for: Phytochromes transmit photoperiod information via the evening complex in Brachypodium
Source: Genome Biol. 2023 Nov 7;24:256. doi: 10.1186/s13059-023-03082-w (PMC10631206; doi:10.1186/s13059-023-03082-w)
Supplement: Supplementary file 10 — Additional file 10: Fig. S1. Phytochromes are necessary for LD activation of flowering. A. phyC-4 does not flower in inductive conditions. B. phyA-1 is late flowering in long days. Fig. S2. Night length determines flowering phenotype in Brachypodium and a night break promotes early flowering A. Plants grown under 12L:4D condition flower nearly at the same time as plants grown und 20L:4D (LD) conditions at after about 3 weeks, whereas plants grown under 12L:12D, 20L:12D or 12L:20D did not flower during the course of the experiment. Experiment was terminated after 75 days, as plants started to senesce. A night break triggers flowering under non inductive short day conditions in Brachypodium. B. Introducing a night break of 1h or 2 nigh breaks of 30min each leads to a flowering phenotype similar to plant being grown under inductive long day conditions (growth condition set up was: 2*1h NB: 12 hour light + 4 hours dark+1hour light+3 hours dark+ 1hour light+3 hours dark. 2*0.5h NB: 12 hour light + 4 hours dark+0.5 hour light+3.5 hours dark+ 0.5 hour light+3.5 hours dark1*1h NB: 12 hour light + 6 hours dark+1hour light+5 hours dark, all Bd21). Fig. S3. Transcriptional and ELF3 bound pattern of representative genes. RPKM was used to show ChIP signal in IGV screenshots. Fig. S4. Transcriptional and ELF3 bound pattern of representative genes. RPKM was used to show ChIP signal in IGV screenshots. Fig. S5. Transcriptional and ELF3 bound pattern of representative genes. RPKM was used to show ChIP signal in IGV screenshots. Fig. S6. Transcriptional and ELF3 bound pattern of representative genes. RPKM was used to show ChIP signal in IGV screenshots. Fig. S7. ppd1-1 transcriptome shows a similar behavior to phyC-4. A. Transcripts were clustered according photoperiod response, same with Fig. 2A, B. Transcriptional pattern of selected genes in Bd21-3, ppd1 and PPD1 OX under SD and LD. Fig. S8. ELF3 protein is degraded in response to light. Independently of photoperiod Plants were gr [file 13059_2023_3082_MOESM10_ESM.pdf]

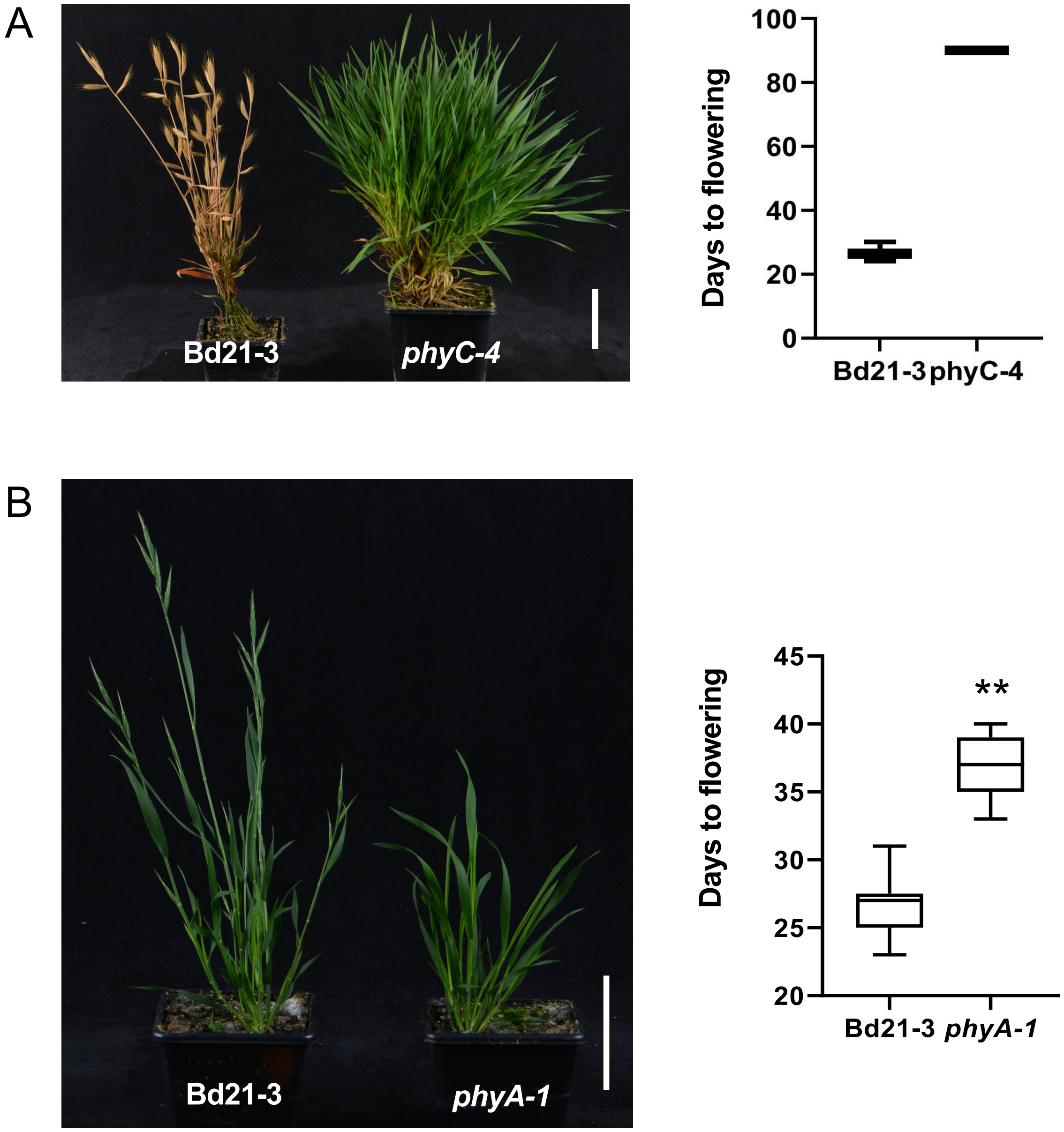

Fig. S1. Phytochromes are necessary for LD activation of flowering.
A. *phyC-4* does not flower in inductive conditions.
B. *phyA-1* is late flowering in long days.

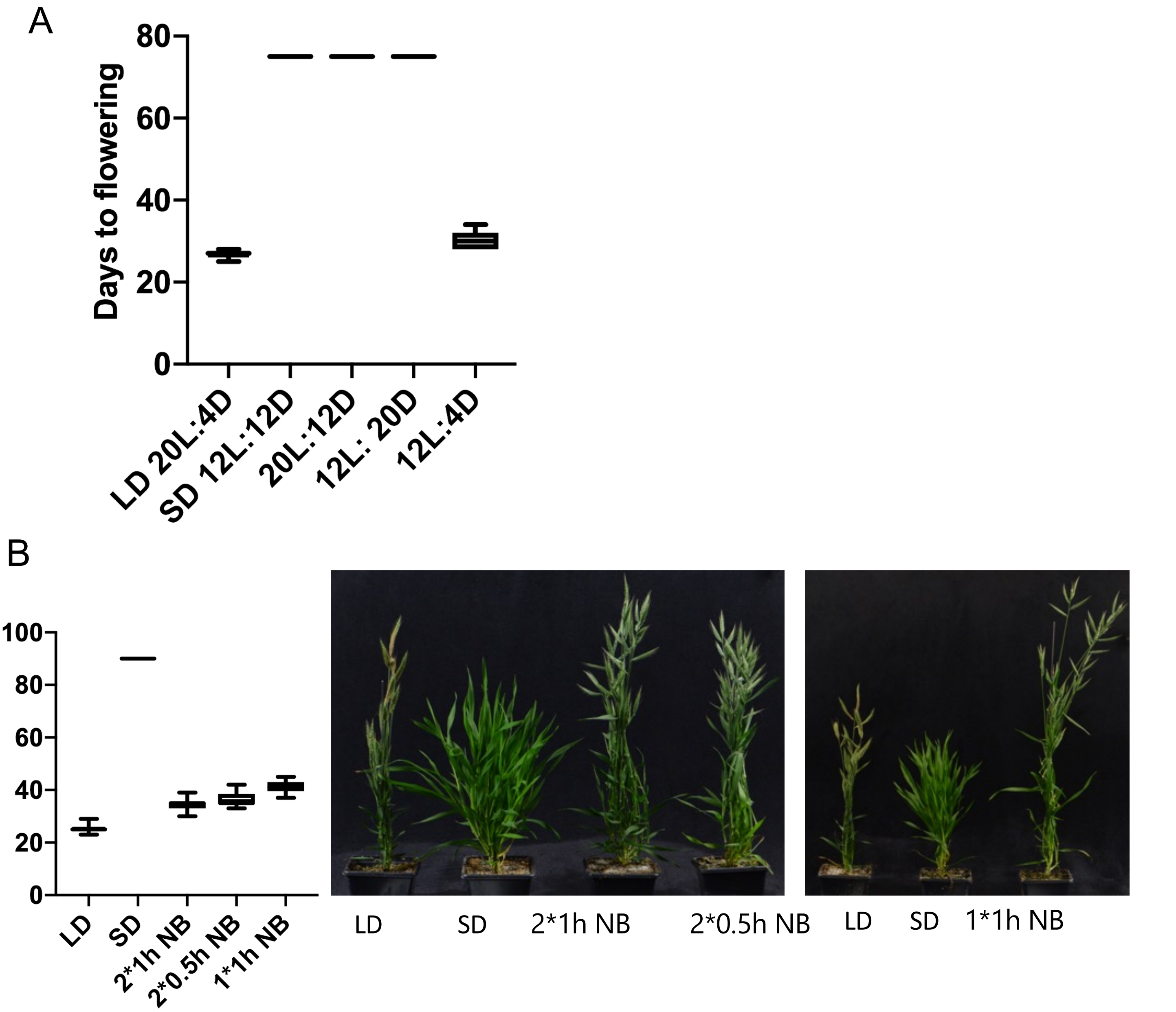

Fig. S2. Night length determines flowering phenotype in *Brachypodium* and a night break promotes early flowering A. Plants grown under 12L:4D condition flower nearly at the same time as plants grown und 20L:4D (LD) conditions at after about 3 weeks, whereas plants grown under 12L:12D, 20L:12D or 12L:20D did not flower during the course of the experiment. Experiment was terminated after 75 days, as plants started to senesce. A night break triggers flowering under non inductive short day conditions in Brachypodium. **B.** Introducing a night break of 1h or 2 nigh breaks of 30min each leads to an flowering phenotype similar to plant being grown under inductive long day conditions (growth condition set up was: 2*1h NB: 12 hour light + 4 hours dark+1hour light+3 hours dark+ 1hour light+3 hours dark. 2*0.5h NB: 12 hour light + 4 hours dark+0.5 hour light+3.5 hours dark+ 0.5 hour light+3.5 hours dark1*1h NB: 12 hour light + 6 hours dark+1hour light+5 hours dark, all Bd21).

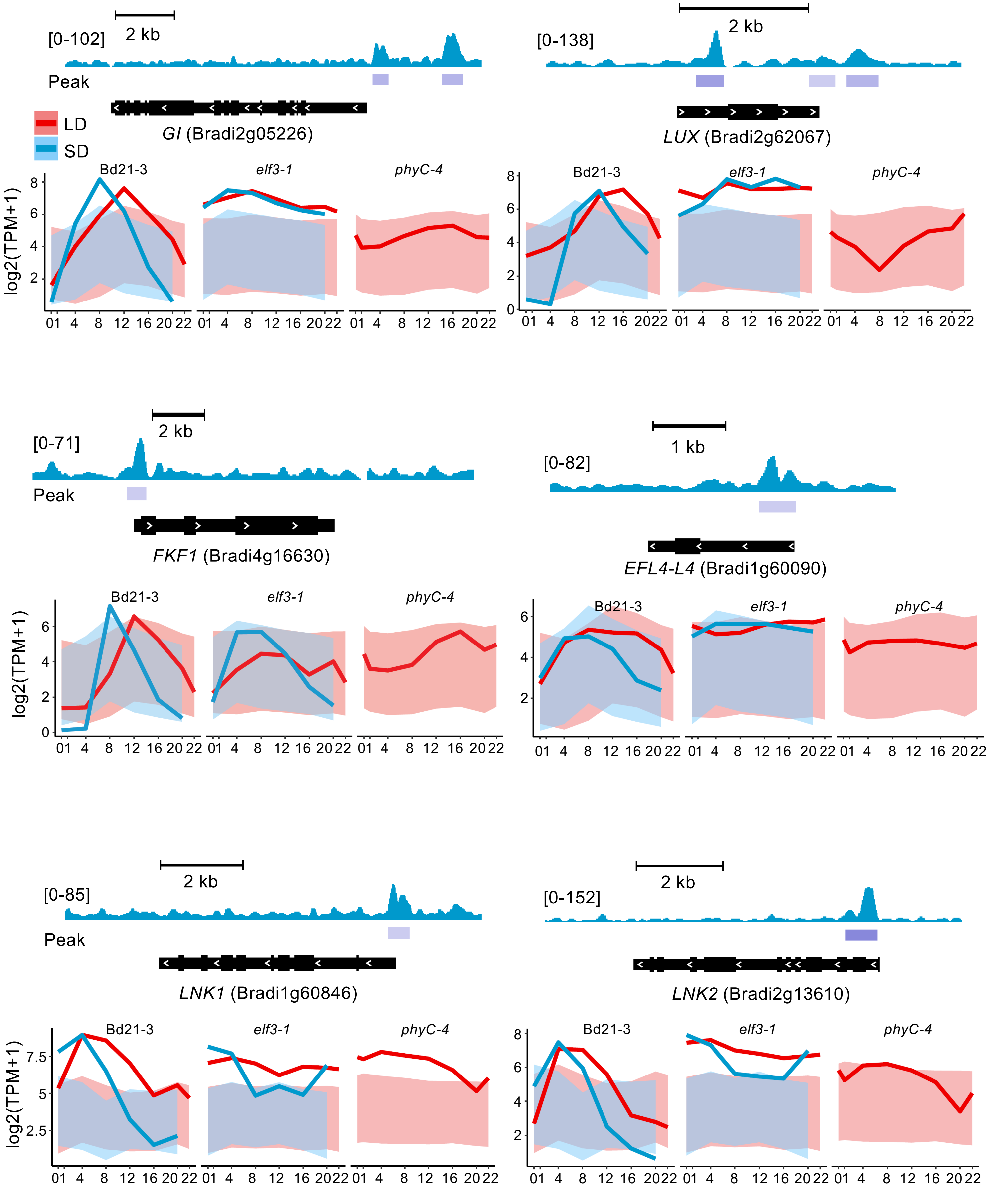

Fig. S3. Transcriptional and ELF3 bound pattern of representative genes. RPKM was used to show ChIP signal in IGV screenshots.

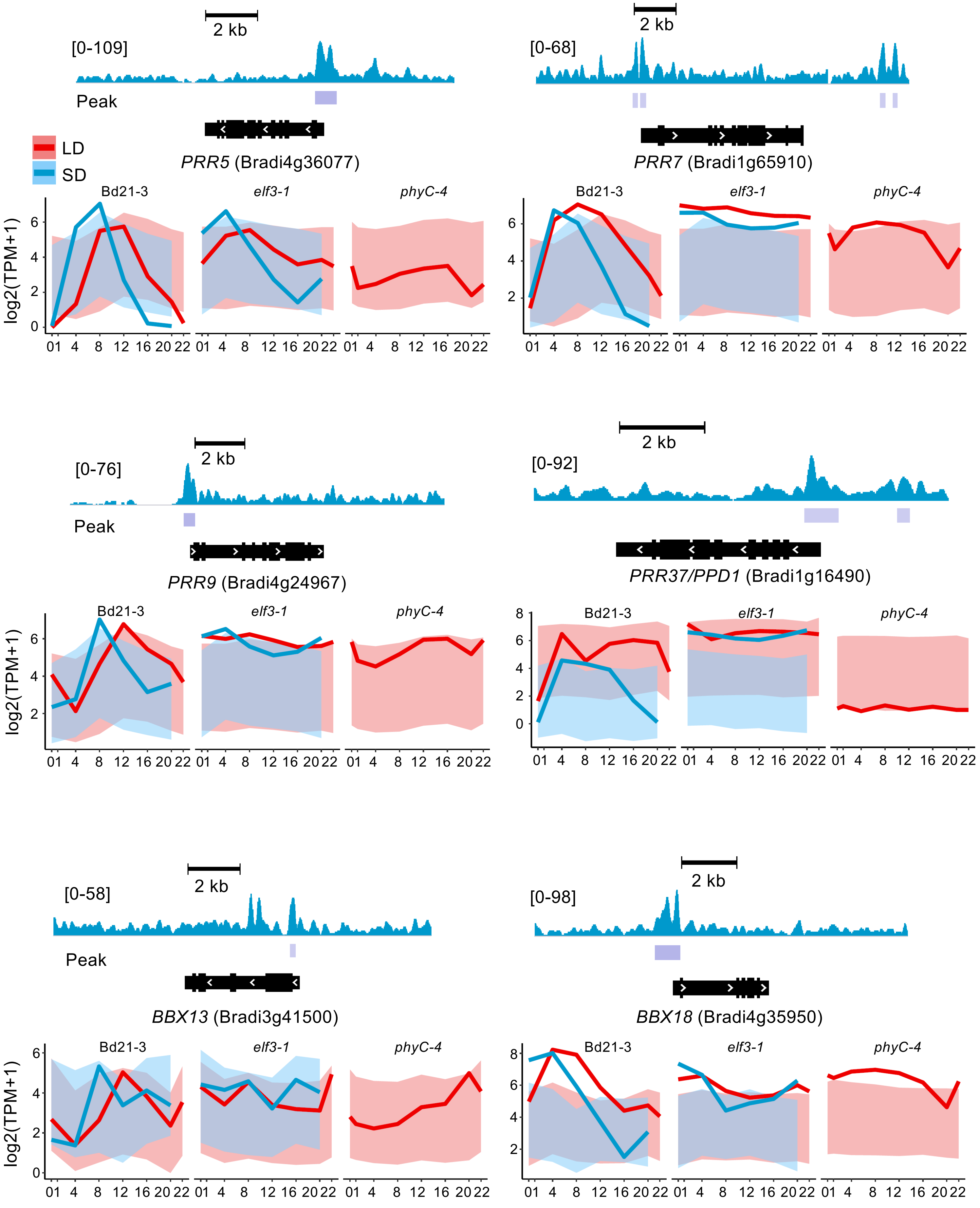

Fig. S4. Transcriptional and ELF3 bound pattern of representative genes. RPKM was used to show ChIP signal in IGV screenshots.

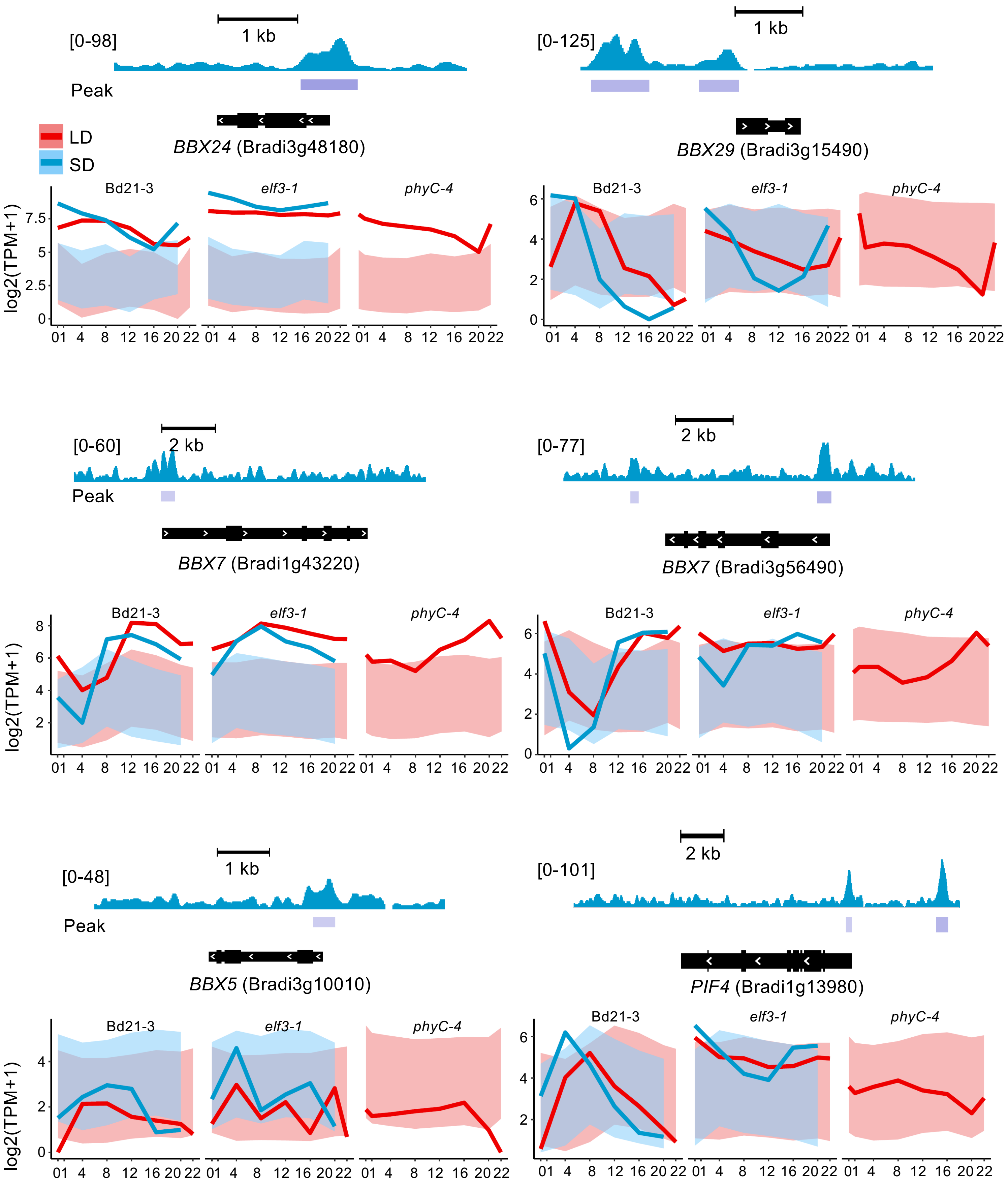

Fig. S5. Transcriptional and ELF3 bound pattern of representative genes. RPKM was used to show ChIP signal in IGV screenshots.

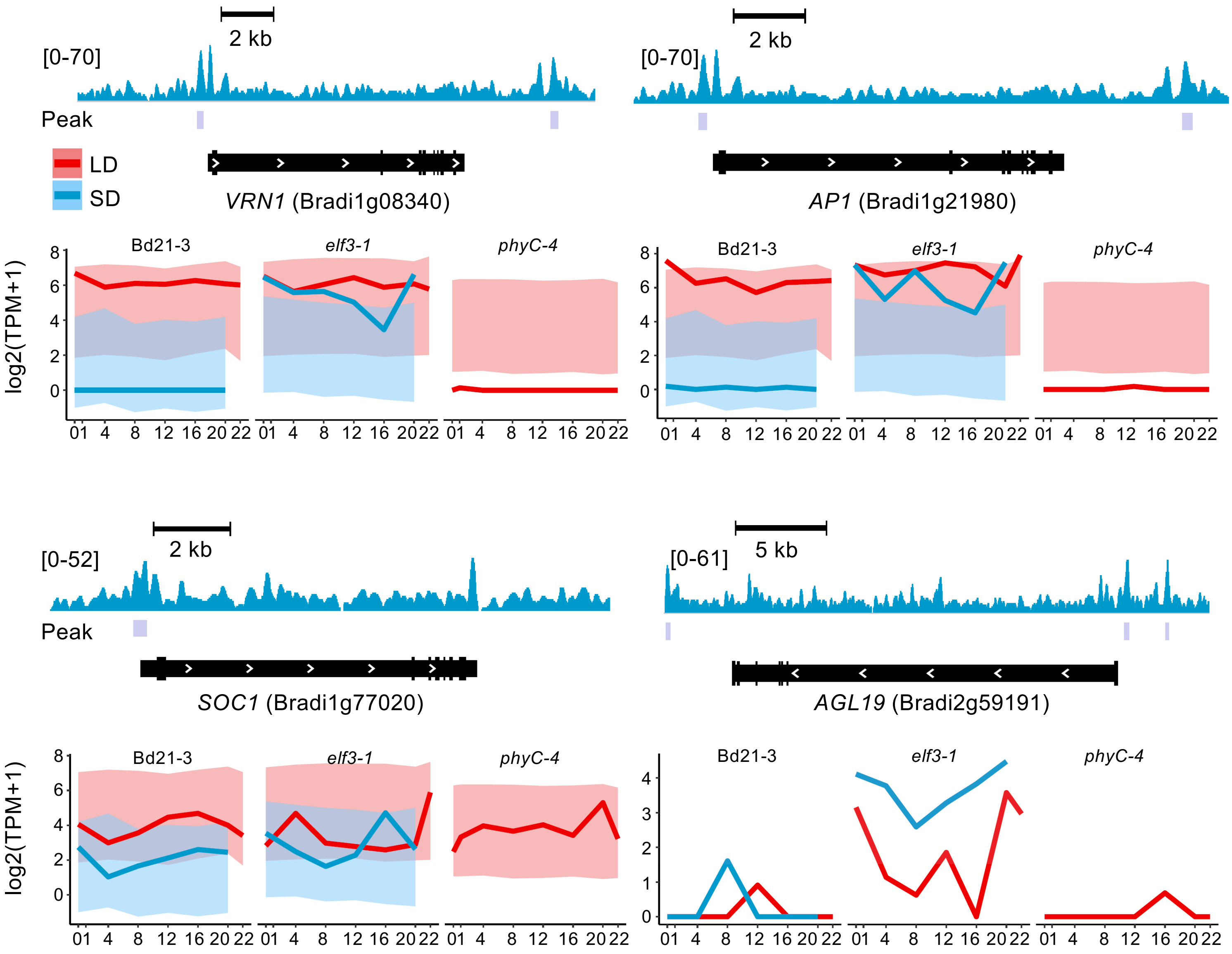

Fig. S6. Transcriptional and ELF3 bound pattern of representative genes. RPKM was used to show ChIP signal in IGV screenshots.

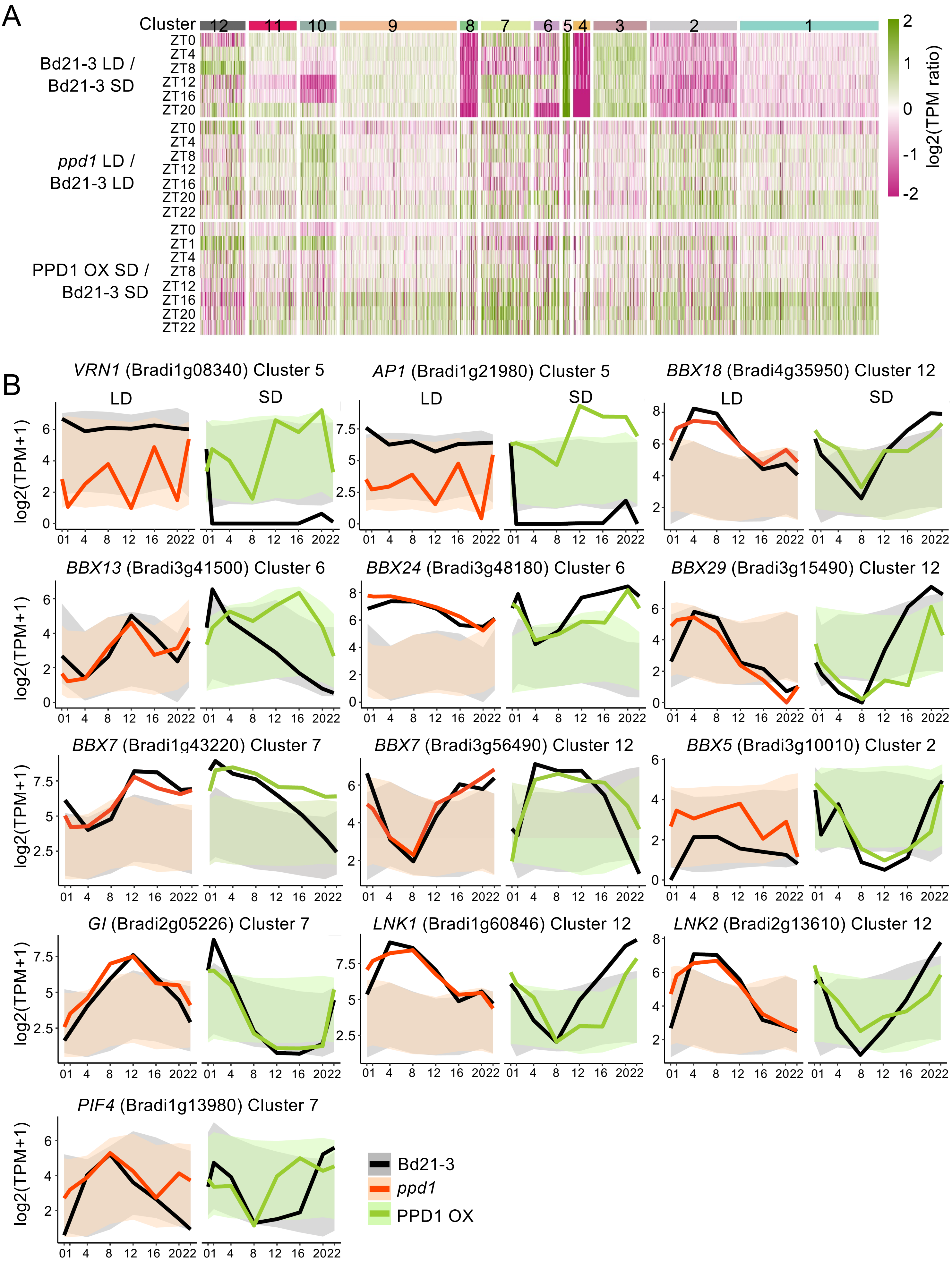

Fig. S7. *ppd1-1* transcriptome shows a similar behavior to *phyC-4*. A. Transcripts were clustered according photoperiod response, same with fig. 2A. B. Transcriptional pattern of selected genes in Bd21-3, *ppd1* and PPD1 OX under SD and LD.

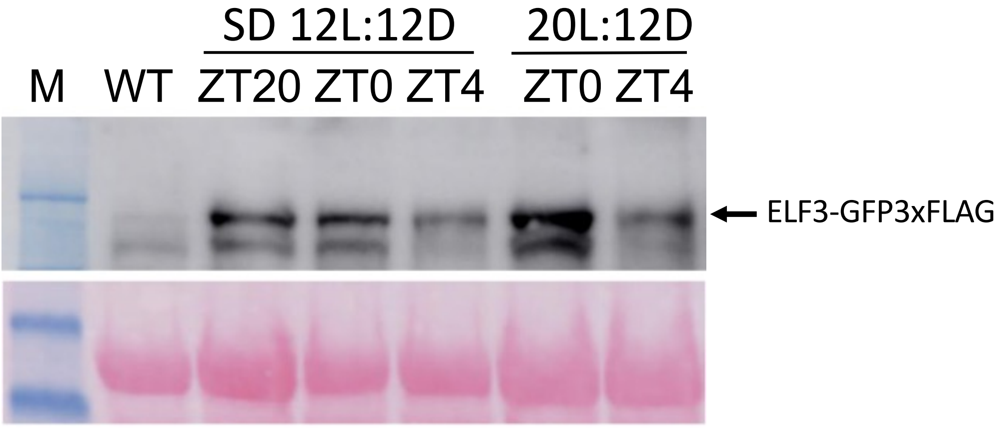

Fig. S8. ELF3 protein is degraded in response to light. Independently of photoperiod

Plants were grown under 12L:12D (SD) or 20L:12D condition as indicated and samples taken 12 DAG at the indicated time (ZT20, ZT0 and ZT4, with 3 plants used per sample). We used *wild type* plants (lane 2, ZT0) or plants overexpressing ELF3 *(pUBI:ELF3_GFP_FLAG)* (lane 3 to lane 7) and probed with an antibody raised in rabbit against ELF3 peptide *(Agrisera AS184168, lot# 1808)*

A

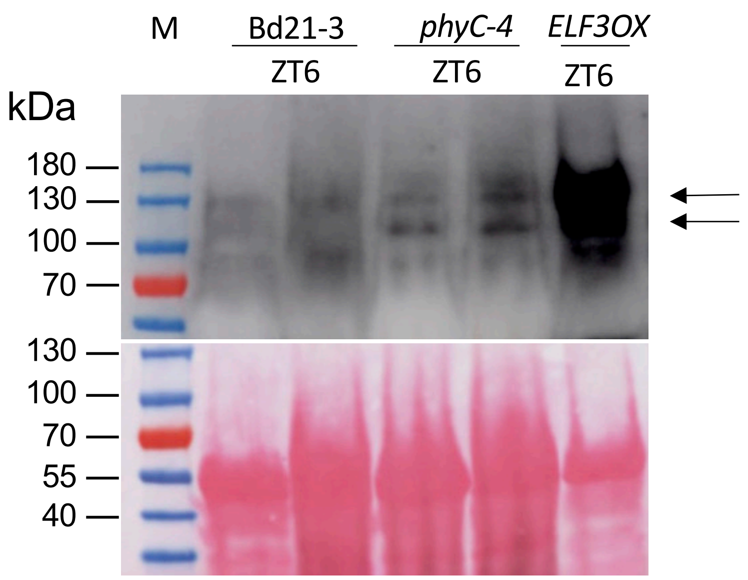

B

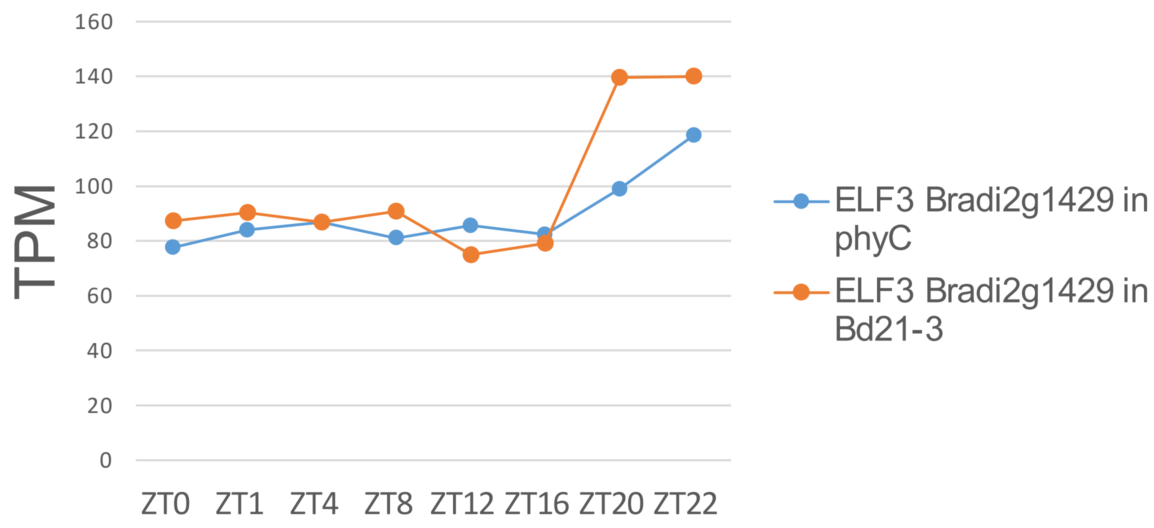

Fig. S9. ELF3 protein is stabilized in *phyC-4*
A. Plants were grown under 20L:12D condition samples taken 12 DAG at the indicated time (ZT6, with 3 plants used per sample). We used Bd21-3 (lane 2, 3), phyC-4 (lane 4, 5) or plants overexpressing ELF3 *(pUBI:ELF3_GFP_FLAG)* (lane 5) and probed with an antibody raised in rabbit against ELF3 peptide *(Agrisera).* ELF3 accumulates in *phyC-4* background at the end of the long day, but can not be detected in Bd21-3 background. ELF3 was detected using custom anti-ELF3 (Agrisera, AS184168, lot# 1808). **B.** Transcript level of ELF3 in phyC background are unchanged, indicating that phyC controls ELF3 in the protein level.

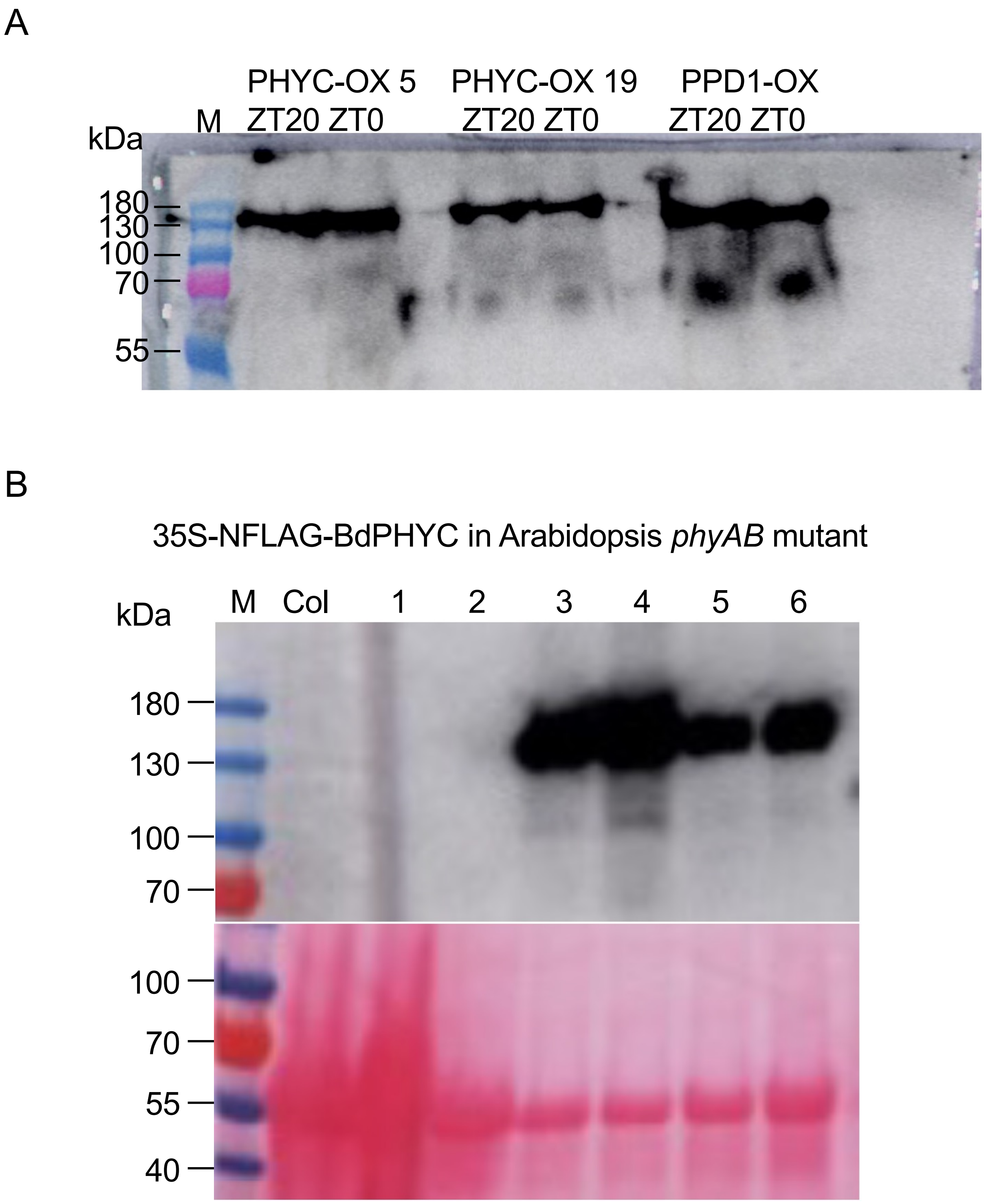

Fig. S10 A. Western blot for 2 independent lines overexpressing pUBI:phyC-GFP-Flag in Brachypodium wild type background. B. Western blot for 6 independent lines overexpressing 35S-NFlag-BdPHYC in Arabidopsis *phyAB* background. Western blot was probed with an antibody against Flag epitope (M2, Sigma). Plants were grown under 20L:4D (LD) condition and samples taken 12 DAG at the indicated time (ZT20, ZT0 with 3 plants used per sample).
