# Supplementary material for: Phytochromes transmit photoperiod information via the evening complex in Brachypodium
Source: Genome Biol. 2023 Nov 7;24:256. doi: 10.1186/s13059-023-03082-w (PMC10631206; doi:10.1186/s13059-023-03082-w)
Supplement: Supplementary file 11 — Additional file 11. Review history. [file 13059_2023_3082_MOESM11_ESM.docx]

**1^st^ round**

**Reviewer 1**

Gao et al., report the role of the photoperiodic responsive protein EARLY FLOWERING3 (ELF3) acts as a floral repressor by interacting phytochrome C (phyC) and directly regulating the expression of some conserved flowering regulators such as PPD1 and VRN1.
-In Fig 7, the authors draft the cartoon showing the regulatory network of phyC and ELF3, of which the phyC seems to repress the expression of ELF3. But there is no experimental data supporting this regulation. While in Table 1, ELF3 was shown to have the interaction with phyC. I suggest to re-prepare the cartoon in case of the mis-leading of the regulation.
-Furthermore, the interaction among ELF3, ELF4L-4 and LUX was indicated in Fig 7. But there is less data validating their interaction as well as their biological function.
-There are some editing errors in this draft. For instance： "Fig. 4. ELF3 directly controls many the flowering regulator PPD1". I suggest the authors to edit it carefully.

**Reviewer 2**

Major Comments:

Prior to publication it will be important to independently confirm the location of the TDNA because many putative mutants turn out to be incorrectly annotated. Furthermore, even if the mutation were within the 5' UTR of PHYB, does this impact PHYB expression? It is important to characterize any allele prior to publication with respect to T-DNA location as well as effects on expression, but it is particularly important in this case because 1) the phyB TDNA line used in this study is only modestly delayed in flowering compared to reports of loss of function mutants in wheat (Pearce et al., 2016) and thus it is possible that this is a very weak allele (or if annotated incorrectly, not a phyB mutant allele) and 2) only a single allele is described in this paper and all conclusions and speculation are based on this one allele. (Also, because this work is based on a single allele with a weak phenotype, it would be ideal to back cross the putative mutant and determined if a distinguishable phenotype completely co-segregated with the T-DNA.)

Related to the weak nature of the phyB allele, the ppd1 allele in this paper also has a very weak effect on flowering whereas another ppd1 allele results in an extreme delay of flowering in inductive long days although this work in biorxiv is not yet published in a peer-reviewed journal (https://doi.org/10.1101/2022.10.11.511813).

More information is needed in the figure legend to help the reader interpret the data shown in Fig 6E. For example, it would be helpful to note what gPHY and YHC stand for? Flowering time was measured as days to flowering up to 100 days yet there is a dotted line above 100 days with additional black dots; thus, report when did these lines flower and when was the experiment concluded? For YHC lines there are plants that flowered around 100 days and some that still failed to flower, why might that be? If the hourglass model was the main mechanism of photoperiodic flowering, would you not expect even more rapid flowering (e.g., plants that mimic LD grown plants) here? Night break experiments also support a coincidence model. It would benefit the reader to discuss other data/studies in temperate grasses that relate to the hourglass model versus the coincidence model.


Minor Comments:

Summary and Introduction:
-change PHOTOPERIOD DEPENDENT1 to PHOTOPERIOD1 which is the convention used for the full name of PPD1 eg Turner et al., 2005, Shaw et al., 2015; Pearce et al., 2017; Campoli et al., 2012. Also change PHOTOPERIOD DEPENDENT1 to PHOTOPERIOD1 during first use in the introduction.

-"Temperate grasses, such as Brachypodium, barley and wheat also induce flowering through the induction of FT-related genes, however there are differences in the signaling pathways activating FT expression." In addition to needing proper punctuation (genes; however, there), a citation is also needed—e.g., Song et al., 2015; Shaw et al., 2020; and/or Fjellheim et al., 2014

-line 39-45 "The major regulator of natural variation in photoperiod responsiveness in barley is the transcriptional regulator PHOTOPERIOD DEPENDENT1 (Hv-Ppd1), first
identified as dominant allele that accelerates flowering under short day (SD)
conditions, making plants photoperiod insensitive [2]." This is not correct. The mapping of PPD1 in barley and in the paper you cited, Turner et al., 2005 came from mapping the recessive late flowering ppd-H1 phenotype under long days. Natural variation of PPD1 in wheat is the result of dominant mutations in this gene resulting in flowering in either long or short days (e.g. Wilhelm et al., 2009).

-Change "Analyses of ppd1 alleles" to "Analyses of PPD1 alleles" the wheat photo-insensitive PPD1 alleles are not loss of function.

- The way various MADS box regulators are referred to can be a bit confusing. For example, Page 5 line 49 "…as is the key floral regulator AP1/VRN1", Page 7 line 5 "VRN1 and the AP1/SOC1 homologs" and Page 8 line 18 "as well as the related genes Bradi1g21980 (related to AP1)". AP1 is compared to several different genes.

-"It has been hypothesized that a photoperiod dependent repressor may bind this 95 bp region in short days to inhibit flowering." It has now been demonstrated via CHIP in wheat that the photoperiod dependent repressor is ELF3 -- Alvarez et al., 2022.

-"The evening complex (EC), an integral component of the circadian clock…" should be the title sentence of the next paragraph.

-line 17 change phyC to PHYC, line 19 change phyC to PHYC, line 21 change phyC to PHYC

Results and Discussion:
- "elf3-1 plants show constitutive early flowering, largely independent of day-length, indicating that ELF3 is necessary for responding to photoperiod" -- you might cite that this was also noted by Bouche et al., 2022. Front Plant Sci. 2022 Jan 6;12:769194. doi: 10.3389/fpls.2021.769194..


- "Another positive regulator of flowering in Brachypodium and Arabidopsis is the clock gene GIGANTEA (GI), and we see this is also directly repressed by ELF3 and becomes
up-regulated under inductive photoperiods in Brachypodium." This needs a citation. To our knowledge there have not been any reports on the function of GI in Brachypodium

- "The floral transition in wheat, barley and Brachypodium is controlled by the key
MADS box transcription factor VRN1 [23,24]"
Citation needed for barley - e.g. Trevaskis 2010 and/or Deng et al., 2015. Also, the first functional analysis of VRN1 in Brachypodium is Woods et al., 2016 and Yan et al., 2003 is the first analysis of VRN1 in wheat.

- "BdVRN1 knockdown lines are late flowering, and higher levels of BdVRN1 expression are associated with early flowering [23]." Ream et al., 2014 described the overexpression of VRN1 and Woods et al., 2016 the knockdown of VRN1 years earlier than reference 23.

- "Phytochromes have been observed to interact with ELF3 in other systems, so this finding is consistent with these observations and suggests phyC may suppress the ability of ELF3 to repress its targets." Recently it has been demonstrated using phyC/elf3 double mutants in Brachypodium that indeed the light signal perceived by PHYs with respect to photoperiodic flowering is mediated by ELF3 not only in other systems, but in Brachypodium as well (Woods et al., 2022). These genetic results in Woods et al., 2022 are consistent with your omic data.

-With regards to the important downstream targets of ELF3 that might impact flowering. It is stated "Since ppd1-1 does not have as strong a flowering
phenotype as phyC-4 this indicates that other directly regulated ELF3 targets
such as the other PRR genes, VRN1 and the AP1/SOC1 homologs, the BBX
genes, GI, LNK1 and 2 and perhaps PIF4 also contribute to the ELF3-photoperiod
flowering response." Recently it has been shown that elf3/ppd1 double mutants under 16h long days are slightly later flowering than ppd1 single mutants demonstrating genetically that PPD1 is a key downstream target of ELF3 with respect to photoperiodic flowering (Woods et al., 2022). Furthermore, the ppd1 mutant used in Woods et al., 2022 has a more delayed flowering phenotype than the CRISPR ppd1 alleles used in this study suggesting that the CRISPR ppd1 mutant used might be a weaker hypomorph.

- "Indeed, ELF3 protein accumulates at the end of the night to high levels under SD, and is rapidly degraded upon exposure to light" at the end of the sentence it should be added that "this is consistent with recent reports in wheat (Alvarez et al., 2022)" because it would be helpful to the readers to draw comparisons with similar work done in Brachypodium, other grasses and Arabidopsis when possible (and throughout the paper you have noted many useful comparisons—this comment provides one more).

- "Phytochromes have been observed to interact with ELF3 in other systems…" Provide a citation - e.g ., Andrade et al., 2022 (rice), Alvarez et al., 2022 (wheat), and Liu et al., 2001 (Arabidopsis)

-Figure 6C-D. Include data either in the main figure or supplement quantifying days to heading and the number of plants used for each experiment.

-In the Materials and Methods describe how the stabilized phyC (YHC) line was generated. The point mutation in phyC is predicted to not undergo dark reversion; was this confirmed or based on other work that should be cited?

- Change "…since night-break experiments perturb flowering in wheat and rice [27,28]" to "since night-break experiments perturb flowering in Brachypodium, wheat, and rice [13, 27,28]

**Authors’ response**

We thank the reviewers for their extremely detailed and helpful feedback. We are grateful for this opportunity to improve the manuscript and thank them for their time and constructive comments.

Reviewer reports:

Reviewer #1: Gao et al., report the role of the photoperiodic responsive protein EARLY FLOWERING3 (ELF3) acts as a floral repressor by interacting phytochrome C (phyC) and directly regulating the expression of some conserved flowering regulators such as PPD1 and VRN1.

1. In Fig 7, the authors draft the cartoon showing the regulatory network of phyC and ELF3, of which the phyC seems to repress the expression of ELF3. But there is no experimental data supporting this regulation. While in Table 1, ELF3 was shown to have the interaction with phyC. I suggest to re-prepare the cartoon in case of the mis- leading of the regulation.

Thank you very much for pointing this out. To clarify, we are proposing that phyC represses the activity of ELF3 protein (indicated by the rectangle with rounded corners). This is fully consistent with the observation that ELF3 is bound by phyC in vivo. We agree with the reviewer that this is an important point. We therefore tested ELF3 expression in phyC background using ELF3 antibody against native ELF3 protein and we observed an accumulation of ELF3 protein phyC during the day (Fig S9 of the new version). Our data therefore indicate that active PHYC directly represses the accumulation of ELF3 protein.

2. Furthermore, the interaction among ELF3, ELF4L-4 and LUX was indicated in Fig 7. But there is less data validating their interaction as well as their biological function.

Thank you very much for your comment. In Table 1 we showed a curated list of proteins co-purified with BdELF3-GFP-FLAG identified from affinity purification coupled with mass spectrometry (AP-MS) among these LUX and ELF4. To further confirm this, we performed a Yeast-2-Hybrid assay, showing the interaction between ELF3 and LUX in vitro (Fig 2A of the new version). We also performed a Split-LUC assay and showed the interaction between ELF3 and LUX in vivo (Fig 2B of new version).

3. There are some editing errors in this draft. For instance： "Fig. 4. ELF3 directly controls many the flowering regulator PPD1". I suggest the authors to edit it carefully.

Thank you very much for pointing this out. This has been corrected.

Reviewer #2: ===

Major Comments:

1. Prior to publication it will be important to independently confirm the location of the TDNA because many putative mutants turn out to be incorrectly annotated.

Furthermore, even if the mutation were within the 5' UTR of PHYB, does this impact PHYB expression? It is important to characterize any allele prior to publication with respect to T-DNA location as well as effects on expression, but it is particularly important in this case because 1) the phyB TDNA line used in this study is only modestly delayed in flowering compared to reports of loss of function mutants in wheat (Pearce et al., 2016) and thus it is possible that this is a very weak allele (or if annotated incorrectly, not a phyB mutant allele) and 2) only a single allele is described in this paper and all conclusions and speculation are based on this one allele. (Also, because this work is based on a single allele with a weak phenotype, it would be ideal to back cross the putative mutant and determined if a distinguishable phenotype completely co-segregated with the T-DNA.)

This is an excellent point. The T-DNA line was obtained from the Brachypodium T-DNA Collection:

https://jgi.doe.gov/our-science/science-programs/plant- genomics/brachypodium/brachypodium-t-dna-collection.

The location of the T-DNA insertion is in the 5’UTR and has been confirmed with genotyping and we could confirm the line to be homozygous. But unfortunately, we couldn’t observe the effects on PHYB expression through real time qPCR. Apart from flowering time experiments, this line has not been used for any further experiments. The phenotype of phyB T-DNA lines are weak and we can’t observe the downregulation of phyB in this T-DNA line. Moreover, this study focuses on the role of phyC. We therefore agree with this reviewer’s comments and have removed the section discussing the phyB mutant.

2. Related to the weak nature of the phyB allele, the ppd1 allele in this paper also has a very weak effect on flowering whereas another ppd1 allele results in an extreme delay of flowering in inductive long days although this work in biorxiv is not yet published in a peer-reviewed journal (https://doi.org/10.1101/2022.10.11.511813).

Thank you very much for your comment. We created ppd1 mutant through CRISPR- Cas9 genome editing method. It showed 1bp deletion in the sixth exon. It flowers significantly later than the wild type control and this is also reflected in our genome expression analysis. As the flowering control was regulated by complicated complex, it is possible that the different allele of ppd1mutant resulting varying degrees of delayed flowering. In our research, ppd1 might be a weaker hypomorph, as mentioned in comment 17.

3. More information is needed in the figure legend to help the reader interpret the data shown in Fig 6E. For example, it would be helpful to note what gPHY and YHC stand for? Flowering time was measured as days to flowering up to 100 days yet there is a dotted line above 100 days with additional black dots; thus, report when did these lines flower and when was the experiment concluded? For YHC lines there are plants that flowered around 100 days and some that still failed to flower, why might that be? If the hourglass model was the main mechanism of photoperiodic flowering, would you not expect even more rapid flowering (e.g., plants that mimic LD grown plants) here? Night break experiments also support a coincidence model. It would benefit the reader to discuss other data/studies in temperate grasses that relate to the hourglass model versus the coincidence model.

Thank you for this observation. We have added more text in the Figure 7E (new version) and also in materials and methods sections and we hope this makes it more clear now

For measuring the flowering days of gPHYC and YHC plants, we grow the plants in the SD chambers and count the flowered plants until 100 days. The additional black dots above 100 days represented the non-flowered plants. As we can see the differences between the gPHYC and YHC plants, we ceased the experiments at 100 days. It is possible that the remaining plants would flower if we waited for longer time. Due to constraints of space, we cannot expand the discussion on hourglass model versus the coincidence model but we are planning to discuss this in a review in more detail.

Minor Comments:

4. Summary and Introduction:

-change PHOTOPERIOD DEPENDENT1 to PHOTOPERIOD1 which is the convention used for the full name of PPD1 eg Turner et al., 2005, Shaw et al., 2015; Pearce et al., 2017; Campoli et al., 2012. Also change PHOTOPERIOD DEPENDENT1 to PHOTOPERIOD1 during first use in the introduction.

We have made these corrections.

5. –“Temperate grasses, such as Brachypodium, barley and wheat also induce flowering through the induction of FT-related genes, however there are differences in the signaling pathways activating FT expression.” In addition to needing proper punctuation (genes; however, there), a citation is also needed—e.g., Song et al., 2015; Shaw et al., 2020; and/or Fjellheim et al., 2014

We have added these references.

Song YH, Shim JS, Kinmonth-Schultz HA, Imaizumi T. Photoperiodic flowering: time measurement mechanisms in leaves. Annu Rev Plant Biol. 2015;66:441-64. doi: 10.1146/annurev-arplant-043014-115555. Epub 2014 Dec 12. PMID: 25534513; PMCID: PMC4414745.

Shaw LM, Li C, Woods DP, Alvarez MA, Lin H, Lau MY, et al. (2020) Epistatic interactions between PHOTOPERIOD1, CONSTANS1 and CONSTANS2 modulate the photoperiodic response in wheat. PLoS Genet 16(7): e1008812. https://doi.org/10.1371/journal.pgen.1008812

Fjellheim S, Boden S, Trevaskis B. The role of seasonal flowering responses in adaptation of grasses to temperate climates. Front Plant Sci. 2014 Aug 29;5:431. doi: 10.3389/fpls.2014.00431. PMID: 25221560; PMCID: PMC4148898.

6. -line 39-45 "The major regulator of natural variation in photoperiod responsiveness in barley is the transcriptional regulator PHOTOPERIOD DEPENDENT1 (Hv-Ppd1), first identified as dominant allele that accelerates flowering under short day (SD) conditions, making plants photoperiod insensitive [2]." This is not correct. The mapping of PPD1 in barley and in the paper you cited, Turner et al., 2005 came from mapping the recessive late flowering ppd-H1 phenotype under long days. Natural variation of PPD1 in wheat is the result of dominant mutations in this gene resulting in flowering in either long or short days (e.g. Wilhelm et al., 2009).

Thank you for this important correction. We have amended the text to fix this error, and included the additional detail about the natural variation of PPD1 in wheat and the additional references.

7. -Change "Analyses of ppd1 alleles" to "Analyses of PPD1 alleles" the wheat photo- insensitive PPD1 alleles are not loss of function.

This has been corrected.

8. - The way various MADS box regulators are referred to can be a bit confusing. For example, Page 5 line 49 "…as is the key floral regulator AP1/VRN1", Page 7 line 5 "VRN1 and the AP1/SOC1 homologs" and Page 8 line 18 "as well as the related genes Bradi1g21980 (related to AP1)". AP1 is compared to several different genes.

Thank you very much for your comment. This has been changed to “We observe direct control of VRN1 by ELF3, as well as the AP1-related genes Bradi1g21980 and Bradi1g08340 (Fig. S6), …”.

9. -"It has been hypothesized that a photoperiod dependent repressor may bind this 95 bp region in short days to inhibit flowering." It has now been demonstrated via CHIP in wheat that the photoperiod dependent repressor is ELF3 -- Alvarez et al., 2022.

We have made this addition, thank you for suggesting it.

Alvarez MA, Li C, Lin H, Joe A, Padilla M, Woods DP, Dubcovsky J. EARLY FLOWERING 3 interactions with PHYTOCHROME B and PHOTOPERIOD1 are critical for the photoperiodic regulation of wheat heading time. PLoS Genet. 2023 May 10;19(5):e1010655. doi: 10.1371/journal.pgen.1010655. PMID: 37163495; PMCID: PMC10171656.

10. -"The evening complex (EC), an integral component of the circadian clock…" should be the title sentence of the next paragraph.

Thank you very much for your comment here, it has been moved to the next paragraph as the title sentence.

11. -line 17 change phyC to PHYC, line 19 change phyC to PHYC, line 21 change phyC to PHYC

Thank you very much for pointing this out. They have been corrected.

Results and Discussion:

12. - "elf3-1 plants show constitutive early flowering, largely independent of day-length, indicating that ELF3 is necessary for responding to photoperiod" -- you might cite that this was also noted by Bouche et al., 2022. Front Plant Sci. 2022 Jan 6;12:769194. doi: 10.3389/fpls.2021.769194..

Thank you very much for your comment here. We cited this reference in the revised manuscript at line 102.

Consistent with the observation in other studies, elf3-1 plants show constitutive early flowering, largely independent of day-length [ref], indicating that ELF3 is necessary for responding to photoperiod (Fig. 1A-C).

Bouché F, Woods DP, Linden J, Li W, Mayer KS, Amasino RM, Périlleux C. EARLY FLOWERING 3 and Photoperiod Sensing in Brachypodium distachyon. Front Plant Sci. 2022 Jan 6;12:769194. doi: 10.3389/fpls.2021.769194. PMID: 35069625; PMCID: PMC8770904.

13. - "Another positive regulator of flowering in Brachypodium and Arabidopsis is the clock gene GIGANTEA (GI), and we see this is also directly repressed by ELF3 and becomes up-regulated under inductive photoperiods in Brachypodium." This needs a citation. To our knowledge there have not been any reports on the function of GI in Brachypodium

We are sorry for this error. It should be in Arabidopsis and it has been corrected at line 179. Thank you very much for pointing out this. We have added a citation.

14. - "The floral transition in wheat, barley and Brachypodium is controlled by the key MADS box transcription factor VRN1 [23,24]" Citation needed for barley - e.g. Trevaskis 2010 and/or Deng et al., 2015. Also, the first functional analysis of VRN1 in Brachypodium is Woods et al., 2016 and Yan et al., 2003 is the first analysis of VRN1 in wheat.

Thank you very much for your comment here. We cited this reference in the revised manuscript at line 188.

We have added these references: The floral transition in wheat, barley and Brachypodium is controlled by the key MADS box transcription factor VRN1 [23,24,ref]. Greenup AG, Sasani S, Oliver SN, Talbot MJ, Dennis ES, Hemming MN, Trevaskis B. ODDSOC2 is a MADS box floral repressor that is down-regulated by vernalization in temperate cereals. Plant Physiol. 2010 Jul;153(3):1062-73. doi: 10.1104/pp.109.152488. Epub 2010 Apr 29. PMID: 20431086; PMCID: PMC2899939. Deng, W., Casao, M., Wang, P. et al. Direct links between the vernalization response and other key traits of cereal crops. Nat Commun 6, 5882 (2015). https://doi.org/10.1038/ncomms6882

Woods DP, Ream TS, Bouché F, Lee J, Thrower N, Wilkerson C, Amasino RM. Establishment of a vernalization requirement in Brachypodium distachyon requires REPRESSOR OF VERNALIZATION1. Proc Natl Acad Sci U S A. 2017 Jun 20;114(25):6623-6628. doi: 10.1073/pnas.1700536114. Epub 2017 Jun 5. PMID: 28584114; PMCID: PMC5488934.

Yan L, Loukoianov A, Tranquilli G, Helguera M, Fahima T, Dubcovsky J. Positional cloning of the wheat vernalization gene VRN1. Proc Natl Acad Sci U S A. 2003 May 13;100(10):6263-8. doi: 10.1073/pnas.0937399100. Epub 2003 May 1. PMID: 12730378; PMCID: PMC156360.

15. - "BdVRN1 knockdown lines are late flowering, and higher levels of BdVRN1 expression are associated with early flowering [23]." Ream et al., 2014 described the overexpression of VRN1 and Woods et al., 2016 the knockdown of VRN1 years earlier than reference 23.

Thank you very much for your comment here. We cited this reference in the revised manuscript at line 190.

We have added these references: BdVRN1 knockdown lines are late flowering, and higher levels of BdVRN1 expression are associated with early flowering [23, ref].

Ream TS, Woods DP, Schwartz CJ, Sanabria CP, Mahoy JA, Walters EM, Kaeppler HF, Amasino RM. Interaction of photoperiod and vernalization determines flowering time of Brachypodium distachyon. Plant Physiol. 2014 Feb;164(2):694-709. doi:

10.1104/pp.113.232678. Epub 2013 Dec 19. PMID: 24357601; PMCID: PMC3912099. Woods DP, Ream TS, Bouché F, Lee J, Thrower N, Wilkerson C, Amasino RM.

Establishment of a vernalization requirement in Brachypodium distachyon requires REPRESSOR OF VERNALIZATION1. Proc Natl Acad Sci U S A. 2017 Jun 20;114(25):6623-6628. doi: 10.1073/pnas.1700536114. Epub 2017 Jun 5. PMID:

28584114; PMCID: PMC5488934.

16. - "Phytochromes have been observed to interact with ELF3 in other systems, so this finding is consistent with these observations and suggests phyC may suppress the ability of ELF3 to repress its targets." Recently it has been demonstrated using phyC/elf3 double mutants in Brachypodium that indeed the light signal perceived by PHYs with respect to photoperiodic flowering is mediated by ELF3 not only in other systems, but in Brachypodium as well (Woods et al., 2022). These genetic results in Woods et al., 2022 are consistent with your omic data.

Thank you very much for your comment here. We cited this reference and made modification in the revised manuscript at line 239-243.

“Phytochromes have been observed to interact with ELF3 in other systems [ref]. And in line with recent genetic results of phyC/elf3 double mutants in Brachypodium, where phyC/elf3 double mutants showed a restored early flowering phenotype from extreme delayed flowering in phyC single mutant [ref].”

Alvarez MA, Li C, Lin H, Joe A, Padilla M, Woods DP, Dubcovsky J. EARLY FLOWERING 3 interactions with PHYTOCHROME B and PHOTOPERIOD1 are critical for the photoperiodic regulation of wheat heading time. PLoS Genet. 2023 May 10;19(5):e1010655. doi: 10.1371/journal.pgen.1010655. PMID: 37163495; PMCID: PMC10171656.

Daniel P. Woods, Weiya Li, Richard Sibout, Mingqin Shao, Debbie Laudencia- Chingcuanco, John P. Vogel, Jorge Dubcovsky, Richard M. Amasino. PHYTOCHROME C regulation of PHOTOPERIOD1 is mediated by EARLY FLOWERING 3 in Brachypodium distachyon. bioRxiv 2022.10.11.511813; doi: https://doi.org/10.1101/2022.10.11.511813

17. -With regards to the important downstream targets of ELF3 that might impact flowering. It is stated "Since ppd1-1 does not have as strong a flowering phenotype as phyC-4 this indicates that other directly regulated ELF3 targets such as the other PRR genes, VRN1 and the AP1/SOC1 homologs, the BBX genes, GI, LNK1 and 2 and perhaps PIF4 also contribute to the ELF3-photoperiod flowering response." Recently it has been shown that elf3/ppd1 double mutants under 16h long days are slightly later flowering than ppd1 single mutants demonstrating genetically that PPD1 is a key downstream target of ELF3 with respect to photoperiodic flowering (Woods et al., 2022). Furthermore, the ppd1 mutant used in Woods et al., 2022 has a more delayed flowering phenotype than the CRISPR ppd1 alleles used in this study suggesting that the CRISPR ppd1 mutant used might be a weaker hypomorph.

Thank you very much for your comment here. We totally agree with you about the reason for different observation of flowering time in ppd1. That might be possible and we have added this observation.

18. - "Indeed, ELF3 protein accumulates at the end of the night to high levels under SD, and is rapidly degraded upon exposure to light" at the end of the sentence it should be added that "this is consistent with recent reports in wheat (Alvarez et al., 2022)" because it would be helpful to the readers to draw comparisons with similar work done in Brachypodium, other grasses and Arabidopsis when possible (and throughout the paper you have noted many useful comparisons—this comment provides one more).

Thank you very much for your comment. We have added this at the end of the

sentence at line 228.

Indeed, ELF3 protein accumulates at the end of the night to high levels under SD, and is rapidly degraded upon exposure to light, which is consistent with recent reports in wheat [ref].

Alvarez MA, Li C, Lin H, Joe A, Padilla M, Woods DP, Dubcovsky J. EARLY FLOWERING 3 interactions with PHYTOCHROME B and PHOTOPERIOD1 are critical for the photoperiodic regulation of wheat heading time. PLoS Genet. 2023 May 10;19(5):e1010655. doi: 10.1371/journal.pgen.1010655. PMID: 37163495; PMCID: PMC10171656.

19. - "Phytochromes have been observed to interact with ELF3 in other systems…"

Provide a citation - e.g ., Andrade et al., 2022 (rice), Alvarez et al., 2022 (wheat), and Liu et al., 2001 (Arabidopsis)

Thanks for this comment. We have added these references.

Liu XL, Covington MF, Fankhauser C, Chory J, Wagner DR. ELF3 encodes a circadian clock-regulated nuclear protein that functions in an Arabidopsis PHYB signal transduction pathway. Plant Cell. 2001 Jun;13(6):1293-304. doi: 10.1105/tpc.13.6.1293. PMID: 11402161; PMCID: PMC135570.

20. -Figure 6C-D. Include data either in the main figure or supplement quantifying days to heading and the number of plants used for each experiment.

Thank you very much for your comment. We have added the dataset as Supplementary Dataset S9.

21. -In the Materials and Methods describe how the stabilized phyC (YHC) line was generated. The point mutation in phyC is predicted to not undergo dark reversion; was this confirmed or based on other work that should be cited?

This has is predicted on the work in Arabidopsis, which is cited (ref. [39]).

22. - Change "…since night-break experiments perturb flowering in wheat and rice [27,28]" to "since night-break experiments perturb flowering in Brachypodium, wheat, and rice [13, 27,28]

Thank you very much for the comment. We have changed this as suggested.

**2^nd^ round**

**Reviewer 2**

The revision has addressed all of the concerns very well.
